# Utilizing the Pentadehydro-Diels–Alder Reaction for Polycyclic Aromatic Compound Synthesis: Diels–Alder-Based Linker Transformation

**DOI:** 10.3390/molecules30071617

**Published:** 2025-04-04

**Authors:** Ying Xia, Qiaofeng Liang, Chenlong Zhu, Bingfeng Sun

**Affiliations:** School of Pharmaceutical Sciences, Nanjing Tech University, 30 South Puzhu Road, Nanjing 211816, China; 202261109022@njtech.edu.cn (Y.X.); 202261109026@njtech.edu.cn (Q.L.)

**Keywords:** Diels–Alder reaction, thioether-tethered tetrayne, pentadehydro-Diels–Alder reaction, traceless tethered strategy

## Abstract

This study introduces a traceless linker strategy for pentadehydro-Diels–Alder (PDDA) cyclization, in which the sulfide/sulfone linker is strategically repurposed as a diene surrogate. As excellent electron-donating dienes, these linkers react with electron-deficient alkenes and alkynes, resulting in a series of highly selective cyclization products. This cascade reaction efficiently integrates the PDDA reaction with linker transformation, formally eliminating the need for permanent structural constraints. By exploiting the intrinsic reactivity of the linker, this strategy offers a robust and versatile approach to constructing complex polycyclic aromatic architectures, providing a powerful tool for organic synthesis.

## 1. Introduction

The Diels–Alder reaction, first described by Otto Diels and Kurt Alder in 1928, is one of the most significant and widely studied transformations in organic chemistry [1,2]. This cycloaddition reaction occurs between a conjugated diene and a dienophile and typically forms a six-membered ring with high regioselectivity when electrophilic-activated alkenes/alkynes react with electron-rich dienes or vice versa. The stereospecificity of the Diels–Alder reaction depends on the specific conditions and substrates involved [3]. Traditionally, the Diels–Alder reaction is a concerted, stereospecific [4+2] cycloaddition. However, stepwise mechanisms involving zwitterionic or diradical intermediates generally lead to lower stereoselectivity. Due to its ability to efficiently construct carbon–carbon bonds with remarkable selectivity, the reaction remains a cornerstone in the synthesis of complex natural products, pharmaceuticals, and functional materials [4,5,6].

In recent years, the Dehydro-Diels–Alder (DDA) reaction has emerged as a significant extension of the classic Diels–Alder cycloaddition, providing new opportunities for the construction of complex cyclic structures [7,8]. This reaction involves the use of dehydrobenzenoid compounds, typically featuring alkynes or other conjugated systems, which undergo a [4+2] cycloaddition with dienophiles, often resulting in strained cyclic products. The Dehydro-Diels–Alder reaction is gaining prominence for its ability to generate novel molecular architectures with enhanced reactivity or unique properties that are challenging to achieve through traditional methods. Among the various variants of the Dehydro-Diels–Alder reaction, the hexadehydro-Diels–Alder (HDDA) [9,10,11] and pentadehydro-Diels–Alder (PDDA) reactions have garnered particular attention [12,13,14,15].

**Scheme 1:**
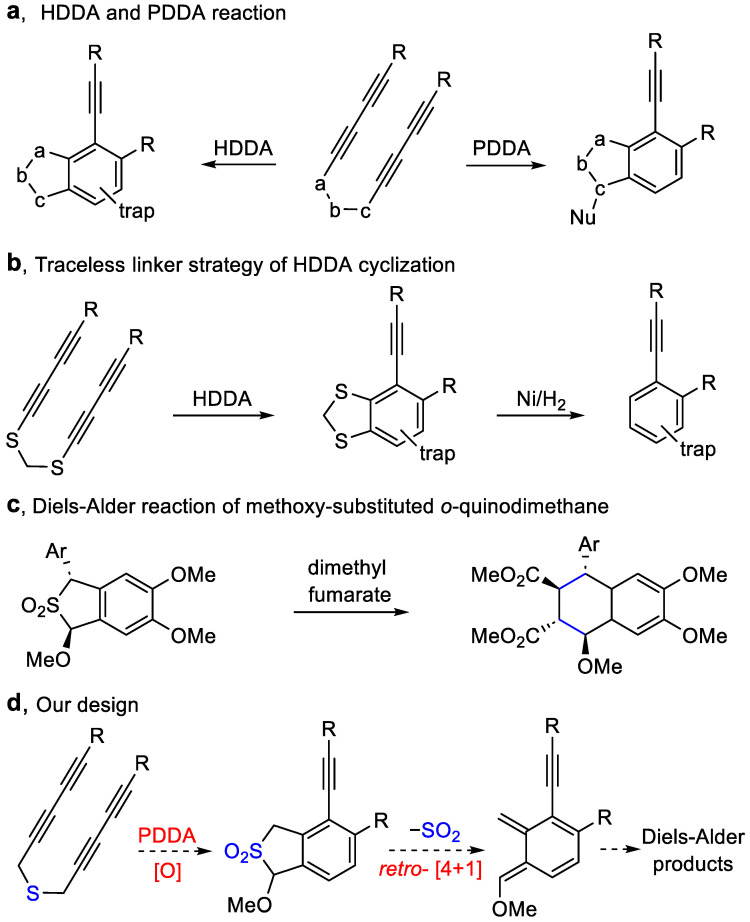
(**a**) HDDA reaction and PDDA reaction, a, b, c represents different atoms. (**b**) Traceless linker strategy of HDDA reaction. (**c**) Diels–Alder reaction between *o*-quinodimethane and dimethyl fumarate. (**d**) Our design: PDDA reaction followed by Diels–Alder based linker transformation.

The HDDA reaction typically involves a three-atom tether linking a diyne and a diynophile, leading to cyclization and the formation of products characterized by a benzannulated linker structure. This strategy has been widely employed in the synthesis of complex polycyclic frameworks, functional materials, and bioactive molecules due to its efficiency in constructing fused aromatic systems (Scheme 1a). The presence of a linker is a defining feature of traditional HDDA reactions, providing structural control and facilitating the formation of regioselective products. In addition to the broad applications of HDDA chemistry, alternative approaches that eliminate the need for linkers or remove them post-cyclization have also been explored [16,17]. Notably, in 2018, the Hoye group [18] reported a sulfur-tethered tetrayne cyclization, in which the HDDA product underwent a desulfurization reaction to afford a linker-free structure. (Scheme 1b) This innovative strategy expands the utility of HDDA reactions by enabling the synthesis of structurally simplified compounds.

Unlike the HDDA reaction, which proceeds via a benzyne intermediate, PDDA operates under basic conditions to generate an allene intermediate that cyclizes and undergoes nucleophilic attack to form the α,3-dehydrotoluenes derivates. While PDDA reactions typically rely on linkers to guide cyclization, linker-free PDDA strategies have not been reported. Inspired by the traceless linker strategy of HDDA reactions, we aim to develop a linker-free PDDA reaction, which could expand the synthetic utility of PDDA transformations and enable new pathways for π-conjugated materials and functionalized molecular architectures.

One example that significantly influenced our conceptualization and design of the project is illustrated in Scheme 1c. Charlton et al. reported that methoxy-*o*-quinodimethanes (*o*-QDMs) react with dimethyl fumarate to yield [4+2] cycloadducts with high yield [19]. Considering the structural characteristics of the PDDA reaction precursor, we designed a thioether-tethered tetrayne substrate (Scheme 1d). First, the α-position of the sulfur atom exhibits high acidity, facilitating deprotonation under basic conditions and thereby promoting PDDA cyclization. Second, the thioether group can be readily oxidized to sulfoxides or sulfones, which, after undergoing PDDA cyclization, generate an *o*-QDM intermediate. This intermediate then reacts with a dienophile, facilitating linker transformation and resulting in the formation of a fused-ring compound.

## 2. Results and Discussion

**Scheme 2:**
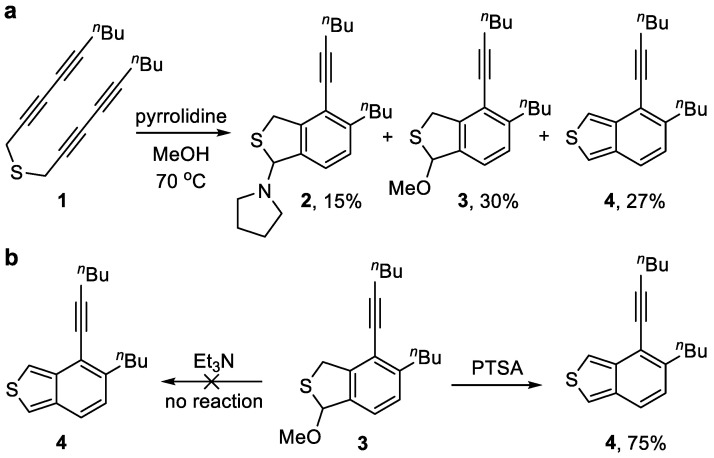
(**a**) Our first example of cyclization of thioether-tethered tetrayne **1** under basic conditions, yielding PDDA products and thiophene. (**b**) Conversion of 1-methoxysulfide **3** to benzo[*c*]thiophene **4**.

With these considerations in mind, we dissolved the thioether-tethered tetrayne in methanol and used pyrrolidine as the base under ambient conditions. As a result, we obtained two additional products: the pyrrolidine adduct **2** with a yield of 15% and the methanol addition product **3** with a yield of 30%. Additionally, we isolated an unstable benzo[*c*]thiophene **4** with a yield of 27%. These results indicate that the PDDA reaction of the thioether-tethered tetrayne under basic conditions is feasible (Scheme 2a). Notably, benzo[*c*]thiophene **4** itself is a diene and serves as an excellent precursor for the Diels–Alder reaction. [20,21] Further investigations revealed that 1-methoxysulfide **3** exhibited stability under basic conditions but underwent 1,4-elimination conversion to thiophene **4** in the presence of *p*-toluenesulfonic acid (PTSA) (Scheme 2b).

To exclude the impact of nucleophilic bases on the reaction, we explored the use of non-nucleophilic bases, including both inorganic and organic types. As shown in Table 1, initially, potassium carbonate (K_2_CO_3_) was examined, and poor conversion of tetrayne **1** was observed when 0.5 equivalents K_2_CO_3_ were used. However, increasing the base loading led to higher isolated yields of cyclization products **3** and **4**, with the product ratio shifting from 4.2:1 to 3.0:1 (entry 2 and 3). This observation suggests that a higher base concentration favors the formation of benzo[*c*]thiophene **4**. Further investigation revealed that cesium carbonate (Cs_2_CO_3_) and potassium *t*-butoxide (*t*-BuOK) exhibited superior performance compared to K_2_CO_3_ and potassium hydroxide (KOH) when used at one equivalent. Notably, employing five equivalents of Cs_2_CO_3_ led to benzo[*c*]thiophene **4** emerging as the major product (entry 6), while using one equivalent of Cs_2_CO_3_ resulted in the highest overall yield among the tested bases (entry 7).

Additionally, we explored the effect of organic bases, including triethylamine (TEA), tetramethylpiperidine (TMP), and 1,8-diazabicyclo[5.4.0]undec-7-ene (DBU). A general trend was observed in which increasing the base loading led to higher overall yields (entries 10–15). When a relatively weak base was used, a higher temperature (100 °C) was required to obtain compounds **3** and **4** in moderate yields (entry 10 and 11). Remarkably, when DBU was employed, the reaction proceeded smoothly, even at room temperature, demonstrating its effectiveness in promoting the transformation under mild conditions.

Another interesting phenomenon was observed: increasing the equivalent of bases, such as K_2_CO_3_, Cs_2_CO_3_, and DBU, reduced the ratio of 1-methoxysulfide **3**, whereas adding an excess amount of base, such as Et_3_N and TMP, can increase the ratio of sulfide **3**. These results suggest that the base plays a crucial role in controlling the reaction products.

**Scheme 3:**
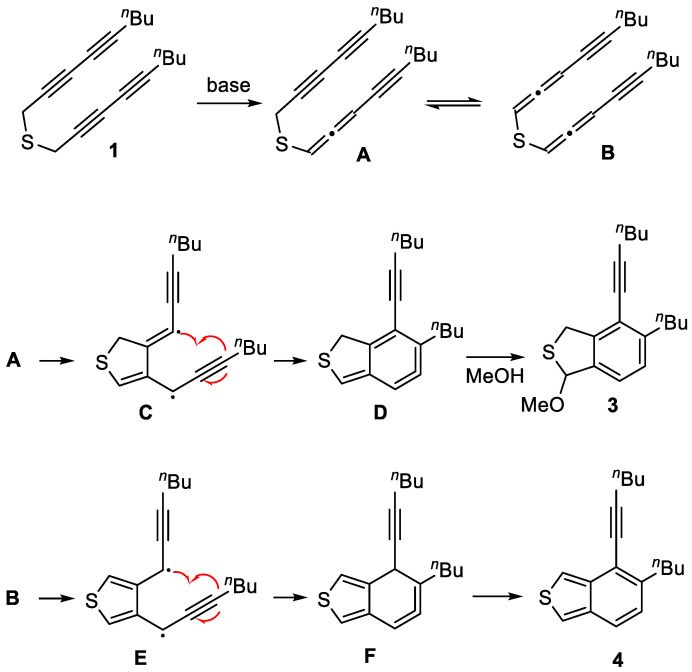
Plausible mechanism for the synthesis of 1-methoxysulfide **3** and benzo[c]thiophene **4** from tetrayne **1**.

Based on literature reports [22,23,24] and the above screening experiments, we propose a plausible mechanism, as depicted in Scheme 3. Under basic conditions, thioether-linked tetrayne **1** undergoes deprotonation to generate allenyne **A** and bis-allenyne **B**. Allenyne **A** subsequently undergoes intramolecular cyclization to furnish diradical species **C**, which then undergoes a nucleophilic addition, leading to the formation of 1-methoxysulfide **3** via intermediate **D**. Meanwhile, bis-allenyne **B** undergoes a simliar transformation pathway, generating diradical intermediate **E**, which undergoes a cyclization event to yield intermediate **F**. Ultimately, a thermodynamically driven isomerization of **F** furnishes benzo[c]thiophene **4** as the final product.

**Scheme 4:**
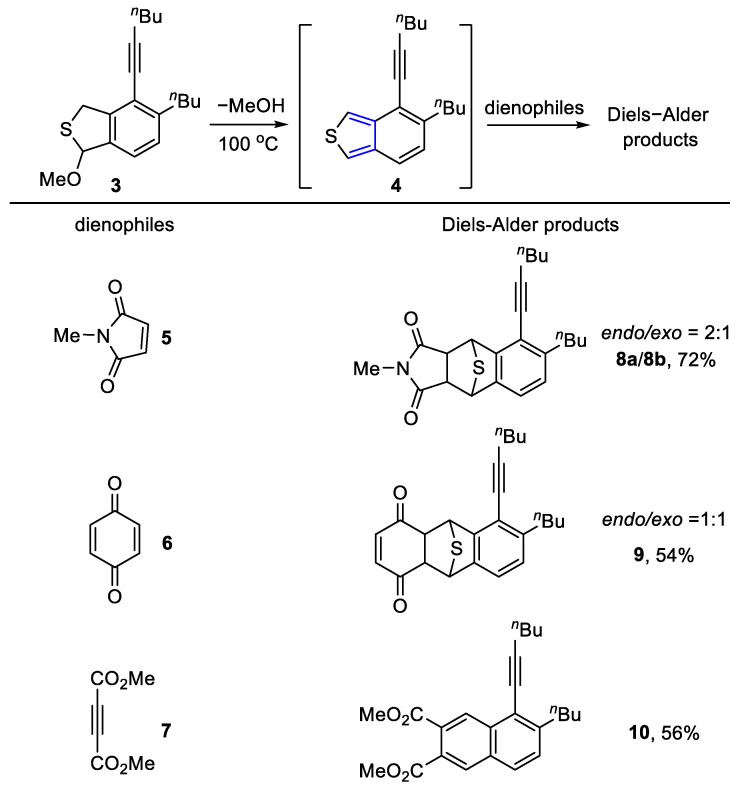
Cycloaddition reactions between 1-methoxysulfide **3** and dienophiles.

With sulfide **3** and **4** in hand, we turned our attention to our primary design. Given that compound **3** could readily convert to **4**, we recognized the potential of benzo[c]thiophene **4** as a highly reactive diene in [4+2] cycloaddition reactions. To explore its reactivity, we selected electron-deficient dienophiles, including *N*-methyl maleimide **5**,

1,4-benzoquinone **6**, and dimethyl acetylenedicarboxylate 7 (DMAD), as reaction partners as shown in Scheme 4. Heating **3** and **5** at 100 °C afforded endo-isomer 8a with a yield of 48% and exo-isomer **8b** with a yield of 24%. The proton chemical shift of the N-methyl group was diagnostic of stereochemistry, with the endo-isomer **8a** being shifted upfield by 0.66 ppm relative to **8b**, owing to the shielding influence of the aryl ring. Therefore, the former and latter isomers were assigned to the endo and exo configurations, respectively. Similarly, the reaction with 1,4-benzoquinone **6** proceeded smoothly, yielding compound **9** as an inseparable mixture of endo and exo isomers in a 1:1 ratio with a moderate yield of 54%. The olefinic protons of one isomer appeared at a higher field (6.07 ppm) than those of the other isomer (6.79 ppm) due to the anisotropic effect of the benzene ring [25]. Therefore, the former was assigned as the endo isomer, while the latter was identified as the exo isomer. Finally, heating **3** with DMAD **7** led to the formation of a Diels–Alder product, followed by desulfurization, affording naphthalene **10** as a single product with a 56% yield.

**Scheme 5:**
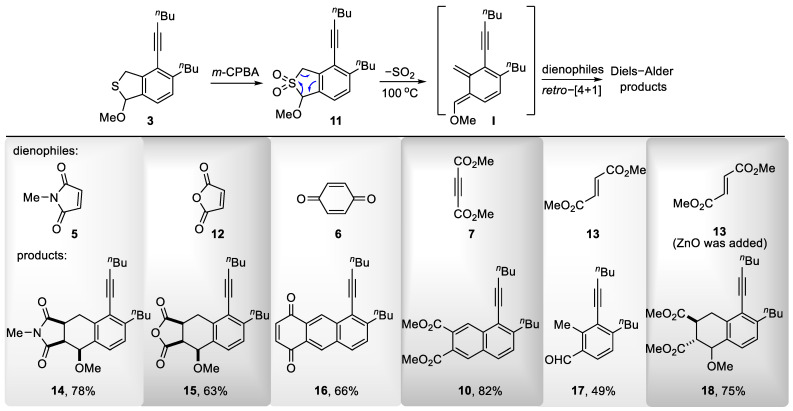
Cycloaddition reactions of 1-methoxysulfone **11** and dienophiles.

Although 1-methoxysulfide **3** can be converted to Diels–Alder adducts with moderate yield, we aim to further improve the conversion efficiency of the linker. Therefore, we revisited the example in Scheme 1c and explored the transformation of l-methoxysulfide **3** into sulfone **11**, followed by its reaction with dienophiles [26]. As depicted in Scheme 5, under epoxidation conditions, sulfide **3** was successfully converted to 1-methoxysulfone **11** with a 77% yield. Subsequently, a retro-[4+1] cycloaddition [27] occurred upon heating, as sulfone **11** underwent SO_2_ extrusion to generate o-quinodimethane **I**, which then reacted with dienophiles, affording the corresponding Diels–Alder adducts. The addition of α-alkoxy o-QDMs to maleic anhydride has been shown to yield only the all-cis endo adducts [28,29]. Similarly, heating sulfone **11** with dienophiles, such as N-methylmaleimide **5** and maleic anhydride **12,** gave all-cis endo adducts **14** and **15** with good yields (78% and 63%, respectively). Treatment of sulfone **11** with benzoquinone **6** yielded the Diels–Alder adduct, followed by aromatization to afford compound **16** with a 66% yield. Reaction with DMAD **7** led to the formation of an elimination and isomerization product **10**, with a higher yield (82%). However, treatment with dimethyl fumarate **13** initially yielded the hydrolysis product **17** at 49%. Notably, the addition of zinc oxide significantly enhanced the yield of cycloadduct **18** as an inseparable isomers with a good yield of 75%, presumably by preventing the acid-catalyzed hydrolysis of intermediate **I** caused by adventitious water.

## 3. Materials and Methods

General: All the chemicals and solvents used for these syntheses were obtained from commercial sources and were used without modification Nuclear magnetic resonance (NMR) spectra (^1^H and ^13^C) were recorded using a Bruker AX-400 spectrometer (Bruker, Bremerhaven, Germany). Chemical shifts in ^1^H NMR spectra recorded in CDCl_3_ are referenced to δ = 7.26 (Data are reported according to the following format: chemical shift (ppm) [multiplicity (e.g., s, d, t, q, etc.), coupling constant(s) (in Hz), integral value (to the nearest whole integer), and structural assignment of the proton]. Chemical shifts in ^13^C NMR spectra are referenced to the carbon atom of CDCl_3_ (δ 77.16). Infrared (IR) spectra were measured on a FT-IR spectrophotometer (Thermo Fisher, Waltham, MA, USA). The samples were placed on a diamond window as thin films (solids by evaporation from a CH_2_Cl_2_ solution and liquids by direct deposition) and recorded in the attenuated total reflectance (ATR) mode. The absorption peak maxima are given in cm^−^^1^. High-resolution mass spectrometry (HRMS) measurements were obtained on a Waters Q-TOF Premier Spectrometer (ESI or EI Source) (Waters Corporation, Milford, MA, USA). Reactions above the ambient laboratory temperature were performed in silicone oil baths that had been pre-equilibrated to the temperature of choice before the reaction vessel was immersed. Column chromatography was generally performed on silica gel (300–400 mesh). Reactions were monitored by thin-layer chromatography (TLC) using UV light to visualize the course of the reactions and an ethanolic solution of phosphomolybdic acid and heat as developing agents. The spectroscopic data of **1**–**4**, **8a**, **8b**, **9**–**11**, **14**–**18** are in Supplementary Materials.

*di(Nona-2,4-diyn-1-yl)sulfane* (**1**)

To a stirred solution of 1-bromonona-2,4-diyne (1.2 g, 6.03 mmol) in MeOH (8 mL) was added sodium sulfide nonahydrate (658 mg, 2.74 mmol). The resulting mixture was stirred at room temperature for 10 min before it was diluted by the addition of H_2_O (5 mL). The layers were separated, and the aqueous layer was extracted with CH_2_Cl_2_ (5 × 10 mL). The combined organic layers were washed with brine (20 mL), dried (Na_2_SO_4_), and concentrated in vacuo. Flash column chromatography (aluminum oxide, hexane) afforded thioether-tethered tetryne 1 (519 mg, 70%) as a reddish brown oil. ^1^H NMR (400 MHz, CDCl_3_): δ 3.48 [s, 4H, S(C*H*_2_)_2_)], 2.27 [t, *J* = 7.4 Hz, 4H, (C≡CC*H*_2_)_2_], 1.57–1.47 [m, 4H, (C≡CCH_2_C*H*_2_)_2_], 1.45–1.36 [m, 4H, (C*H*_2_CH_3_)_2_], and 0.91 [t, *J* = 7.2 Hz, 6H, (CH_2_C*H*_3_)_2_]. ^13^C NMR (101 MHz, CDCl_3_): δ 80.3 (2C), 71.0 (2C), 68.5 (2C), 64.8 (2C), 30.3 (2C), 22.0 (2C), 20.0 (2C), 19.0 (2C), and 13.6 (2C). HRMS (ESI) *m*/*z*: [M + H]^+^ Calcd for C_18_H_23_S^+^ 271.1515; Found 271.1519. IR (film): 2954, 2255 (C≡C), 1158, 1073, and 856 cm^−1^.

*1-(5-Butyl-4-(hex-1-yn-1-yl)-1,3-dihydrobenzo[c]thiophen-1-yl)pyrrolidine* (**2**)

To a stirred solution of thioether **1** (386 mg, 1.43 mmol) in MeOH (5 mL) was added pyrrolidine (1.20 mL, 14.27 mmol). The resulting mixture was stirred at 70 °C for 4 h before it was quenched by the addition of NH_4_Cl (10 mL, satd aq). The layers were separated, and the aqueous layer was extracted with CH_2_Cl_2_ (3 × 10 mL). The combined organic layers were washed with brine (10 mL), dried (Na_2_SO_4_), and concentrated in vacuo. Flash column chromatography (silica gel, hexanes:EtOAc = 20:1) to afford benzo[*c*]thiophene **4** (104 mg, 27%) as a yellow oil, followed by the slower eluting **3** (130 mg, 30%) as a brown oil, and the pyrrolidine adduct 2 (74 mg, 15%) as a yellow oil. ^1^H NMR (400 MHz, CDCl_3_): δ 7.23 (d, *J* = 7.8 Hz, 1H, Ar*H*), 7.08 (d, *J* = 7.9 Hz, 1H, Ar*H*), 6.32 (d, *J* = 1.9 Hz, 1H, NC*H*), 4.23–4.12 (m, 2H, SC*H*_2_), 2.85–2.70 (m, 2H, ArC*H*_2_), 2.70–2.65 (m, H, NC*H*_2_), 2.48 (t, *J* = 6.8 Hz, 2H, C≡CC*H*_2_), 2.36–2.26 (m, 2H NC*H*_2_), 1.78–1.72 [m, 4H, N(CH_2_C*H*_2_)_2_], 1.63–1.56 [m, 4H, (C*H*_2_CH_2_CH_3_)_2_], 1.54–1.47 (m, 2H, (CH_2_C*H*_2_CH_3_), 1.40–1.33 (m, 2H, CH_2_C*H*_2_CH_3_), and 0.95 [q, *J* = 7.4 Hz, 6H, (CH_2_CH_2_C*H*_3_)_2_]. ^13^C NMR (101 MHz, CDCl_3_): δ 144.7, 143.2, 138.9, 127.6, 124.6, 120.2, 98.8, 80.9, 77.2, 48.8, 36.9, 34.1, 33.0, 31.1, 23.7, 22.7, 22.1, 19.5, 14.1, and 13.8. HRMS (ESI) *m*/*z*: [M + H]^+^ Calcd for C_22_H_32_NS^+^ 342.2250; Found 342.2247. IR (film): 3449, 2261 (C≡C), 1506 (Ar), 1227, and 1159 cm^−1^.

*5-Butyl-4-(hex-1-yn-1-yl)-1-methoxy-1,3-dihydrobenzo[c]thiophene* (**3**)

^1^H NMR (400 MHz, CDCl_3_): δ 7.26 (d, *J* = 7.7 Hz, 1H, Ar*H*), 7.16 (d, *J* = 7.5 Hz, 1H, Ar*H*), 6.44 (q, *J* = 2.3 Hz, 1H, CH_3_OC*H*), 4.38 (d, *J* = 15.1 Hz, 1H, SC*H_a_*H_b_), 4.21 (d, *J* = 15.6 Hz, 1H, SCH_a_*H_b_*), 3.30 (s, 3H, OC*H*_3_), 2.81 (t, *J* = 7.5 Hz, 2H, ArC*H*_2_), 2.52 (td, *J* = 7.1, 2.6 Hz, 2H, C≡CC*H*_2_), 1.65–1.60 [m, 4H, (C*H*_2_CH_2_CH_3_)_2_], 1.57–1.51 (m, 2H, CH_2_C*H*_2_CH_3_), 1.43–1.36 (m, 2H, CH_2_C*H*_2_CH_3_), and 0.98 [q, *J* = 7.7 Hz, 6H, (CH_2_CH_2_C*H*_3_)_2_]. ^13^C NMR (101 MHz, CDCl_3_): δ 145.6, 143.7, 137.5, 128.1, 124.1, 120.4, 99.1, 94.4, 77.0, 53.9, 37.4, 34.1, 33.0, 31.0, 22.7, 22.1, 19.4, 14.1, and 13.7. HRMS (ESI) *m*/*z*: [M + H]^+^ Calcd for C_19_H_27_OS^+^ 303.1777; Found 303.1780. IR (film): 3473, 2090 (C≡C), 1640, 1465 (Ar), and 1186 cm^−1^.

*5-Butyl-4-(hex-1-yn-1-yl)benzo[c]thiophene* (**4**)

^1^H NMR (400 MHz, CDCl_3_): δ 7.75 (dd, *J* = 3.4, 1.1 Hz, 1H, SC*H*=C), 7.61 (d, *J* = 3.4 Hz, 1H, SC*H*=C), 7.45 (dd, *J* = 8.9, 1.1 Hz, 1H, Ar*H*), 6.93 (d, *J* = 8.9 Hz, 1H, Ar*H*), 2.85–2.81 (m, 2H, ArC*H*_2_), 2.58 (t, *J* = 6.9 Hz, 2H, C≡CC*H*_2_), 1.72–1.65 (m, 2H, C*H*_2_CH_2_CH_3_), 1.61–1.55 [m, 4H, (CH_2_C*H*_2_CH_3_)_2_], 1.42–1.37 (m, 2H, CH_2_C*H*_2_CH_3_), and 1.01–0.93 [m, 6H, (CH_2_CH_2_C*H*_3_)_2_].

*(3aS,4R,9S,9aR)-6-Butyl-5-(hex-1-yn-1-yl)-2-methyl-3a,4,9,9a-tetrahydro-1H-4,9-epithiobenzo[f]isoindole-1,3(2H)-dione* (**8a**)

To a stirred solution of thioether 3 (120 mg, 0.40 mmol) in chlorobenzene (3 mL) was added *N*-methylmaleimide 5 (220 mg, 1.98 mmol). The resulting mixture was stirred at 70 °C for 1 h before it was quenched by the addition of NaHCO_3_ (10 mL, satd aq). The layers were separated, and the aqueous layer was extracted with CH_2_Cl_2_ (3 × 10 mL). The combined organic layers were washed with brine (10 mL), dried (Na_2_SO_4_), and concentrated in vacuo. Flash column chromatography (silica gel, hexanes:EtOAc = 10:1) to afford compound 8a (72 mg, 48%) as a light yellow oil and followed by the slower eluting 8b (36 mg, 24%) as a light yellow oil. ^1^H NMR (400 MHz, CDCl_3_): δ 7.00 (d, *J* = 7.4 Hz, 1H, Ar*H*), 6.85 (d, *J* = 7.5 Hz, 1H, Ar*H*), 5.11 (t, *J* = 1.4 Hz, 1H, S*H_a_*), 4.82 (t, *J* = 1.3 Hz, 1H, S*H_b_*), 3.28 (dd, *J* = 6.5, 1.4 Hz, 1H, COC*H_a_*), 3.24 (dd, *J* = 6.6, 1.4 Hz, 1H, COC*H_b_*), 3.03 (s, 3H, NC*H*_3_), 2.76–2.59 (m, 2H, ArC*H*_2_), 2.52 (t, *J* = 6.9 Hz, 2H, C≡CC*H*_2_), 1.68–1.60 (m, 2H, C*H*_2_CH_2_CH_3_), 1.59–1.49 [m, 4H, (CH_2_C*H*_2_CH_3_)_2_], 1.41–1.30 (m, 2H, CH_2_C*H*_2_CH_3_), and 0.95 [dt, *J* = 23.1, 7.3 Hz, 6H, (CH_2_CH_2_C*H*_3_)_2_]. ^13^C NMR (101 MHz, CDCl_3_): δ 176.2, 176.0, 148.2, 143.5, 143.3, 126.6, 118.8, 116.7, 98.4, 75.8, 55.9, 55.1, 51.7, 51.0, 34.3, 32.9, 30.9, 25.1, 22.7, 22.1, 19.5, 14.0, and 13.7. HRMS (ESI) *m*/*z*: [M + H]^+^ Calcd for C_23_H_28_NO_2_S^+^ 382.1835; Found 382.1836. IR (film): 3450, 1705 (C=O), 1643, 1456 (Ar), and 1109 cm^−1^.

*(3aR,4R,9S,9aS)-6-Butyl-5-(hex-1-yn-1-yl)-2-methyl-3a,4,9,9a-tetrahydro-1H-4,9-epithiobenzo[f]isoindole-1,3(2H)-dione* (**8b**)

^1^H NMR (400 MHz, CDCl_3_): δ 6.93 (d, *J* = 7.5 Hz, 1H, Ar*H*), 6.84 (d, *J* = 7.6 Hz, 1H, Ar*H*), 5.16 (dd, *J* = 4.2, 1.4 Hz, 1H, S*H_a_*), 4.82 (dd, *J* = 4.1, 1.4 Hz, 1H, S*H_b_*), 4.09–3.95 [m, 2H, (COC*H*)_2_], 2.70–2.60 (m, 2H, ArC*H*_2_), 2.52 (t, *J* = 7.0 Hz, 2H, C≡CC*H*_2_), 2.37 (s, 3H, NC*H*_3_), 1.70–1.61 (m, 2H, C*H*_2_CH_2_CH_3_), 1.57–1.46 [m, 4H (CH_2_C*H*_2_CH_3_)_2_], 1.34–1.24 (m, 2H, CH_2_C*H*_2_CH_3_), and 0.93 [dt, *J* = 32.6, 7.4 Hz, 6H, (CH_2_CH_2_C*H*_3_)_2_]. ^13^C NMR (101 MHz, CDCl_3_): δ 174.8, 173.7, 144.9, 143.9, 139.8, 127.2, 120.1, 117.6, 98.1, 75.8, 55.3, 54.6, 53.2, 52.9, 34.2, 33.0, 31.1, 24.1, 22.5, 22.2, 19.6, 14.0, and 13.8. HRMS (ESI) *m*/*z*: [M + H]^+^ Calcd for C_23_H_28_NO_2_S^+^ 382.1835; Found 382.1834. IR (film): 3461, 2114 (C≡C), 1706 (C=O), 1644, and 1113 cm^−1^.

*6-Butyl-5-(hex-1-yn-1-yl)-4a,9,9a,10-tetrahydro-9,10-epithioanthracene-1,4-dione* (**9**)

To a stirred solution of thioether 3 (128 mg, 0.42 mmol) in chlorobenzene (3 mL) was added 1,4-benzoquinone 6 (137 mg, 1.27 mmol). The resulting mixture was stirred at 100 °C for 5 h before it was quenched by the addition of NaHCO_3_ (10 mL, satd aq). The layers were separated, and the aqueous layer was extracted with CH_2_Cl_2_ (3 × 10 mL). The combined organic layers were washed with brine (10 mL), dried (Na_2_SO_4_), and concentrated in vacuo. Flash column chromatography (silica gel, hexanes:EtOAc = 10:1) afforded 9 (86 mg, *endo* and *exo* 1:1, 54%) as a bright yellow oil. ^1^H NMR (400 MHz, CDCl_3_): δ 7.05 (d, *J* = 7.6 Hz, 1H, COC*H*=CH), 6.86 (m, 2H, Ar*H*), 6.85 (d, *J* = 7.8 Hz, 1H, COC*H*=CH), 6.79 (d, *J* = 7.5 Hz, 2H, Ar*H*), 6.07 [q, *J* = 9.7 Hz, 2H, (COCH=C*H*)_2_], 5.32–5.30 (m, 1H, S*H*), 5.29 (dd, *J* = 3.6, 1.6 Hz, 1H, S*H*), 5.01–4.99 (m, 1H, S*H*), 4.95 (dd, *J* = 3.6, 1.6 Hz, 1H, S*H*), 3.82 (dd, *J* = 7.5, 3.6 Hz, 2H, COC*H*), 3.25 (dd, *J* = 4.4, 1.0 Hz, 2H, COC*H*), 2.75–2.58 [m, 4H, (ArC*H*_2_)_2_], 2.57–2.48 [m, 4H, (C≡CC*H*_2_*)*_2_], 1.69–1.64 [m, 4H, (C*H*_2_CH_2_CH_3_)_2_], 1.59–1.52 [m, 4H, (C*H*_2_CH_2_CH_3_)_2_], 1.49–1.44 [m, 4H, (CH_2_C*H*_2_CH_3_)_2_], 1.38–1.24 [m, 4H, (CH_2_C*H*_2_CH_3_)_2_], 0.99 [td, *J* = 7.3, 3.7 Hz, 6H, CH_2_CH_2_C*H*_3_)_2_], and 0.91 [dt, *J* = 15.9, 7.2 Hz, 6H, (CH_2_CH_2_C*H*_3_)_2_]. ^13^C NMR (101 MHz, CDCl_3_): δ 197.4, 196.4, 195.9, 195.5, 148.5, 145.9, 143.8, 143.7, 143.1, 142.6, 142.2, 140.9, 139.6, 139.0, 127.0, 126.5, 120.0, 118.5, 117.5, 116.3, 98.4, 98.3, 75.9, 75.5, 58.9, 58.7, 58.0, 57.9, 54.0, 53.4, 53.2, 53.0, 34.3, 34.2, 33.03, 32.97,31.00, 30.97, 22.7, 22.5, 22.20, 22.16, 19.50, 19.45, 14.1, 14.0, and 13.78. HRMS (ESI) *m*/*z*: [M + H]^+^ Calcd for C_24_H_27_O_2_S^+^ 379.1726; Found 379.1728. IR (film): 3446, 2089 (C≡C), 1672 (C=O), 1457 (Ar), and 1054 cm^−1^.

*Dimethyl 6-butyl-5-(hex-1-yn-1-yl)naphthalene-2,3-dicarboxylate* (**10**)

To a stirred solution of thioether 3 (135 mg, 0.45 mmol) in chlorobenzene (3 mL) was added dimethyl acetylenedicarboxylate 7 (1.10 mL, 8.93 mmol). The resulting mixture was stirred at 100 °C for 2 h before it was quenched by the addition of NaHCO_3_ (10 mL, satd aq). The layers were separated, and the aqueous layer was extracted with CH_2_Cl_2_ (3 × 10 mL). The combined organic layers were washed with brine (10 mL), dried (Na_2_SO_4_), and concentrated in vacuo. Flash column chromatography (silica gel, hexanes:EtOAc = 10:1) afforded 10 (95 mg, 56%) as a yellow oil. ^1^H NMR (400 MHz, CDCl_3_): δ 8.69 (s, 1H, Ar*H*), 8.21 (s, 1H, Ar*H*), 7.75 (d, *J* = 8.4 Hz, 1H, Ar*H*), 7.46 (d, *J* = 8.5 Hz, 1H, Ar*H*), 3.97 (s, 3H, CO_2_C*H*_3_), 3.95 (s, 3H, CO_2_C*H*_3_), 3.01–2.93 (m, 2H, ArC*H*_2_), 2.63 (t, *J* = 6.9 Hz, 2H, C≡CC*H*_2_), 1.75–1.65 [m, 4H, (C*H*_2_CH_2_CH_3_)_2_], 1.62–1.56 (m, 2H, CH_2_C*H*_2_CH_3_), 1.44–1.37 (m, 2H, CH_2_C*H*_2_CH_3_), and 0.98 [dt, *J* = 18.0, 7.4 Hz, 6H, (CH_2_CH_2_C*H*_3_)_2_]. ^13^C NMR (101 MHz, CDCl_3_): δ 168.8, 168.2, 146.5, 134.3, 131.8, 130.5, 130.1, 129.2, 128.6, 127.8, 127.7, 121.3, 101.1, 76.4, 52.8 (2C), 35.2, 32.9, 31.0, 22.8, 22.2, 19.7, 14.1, and 13.8. HRMS (ESI) *m*/*z*: [M + H]^+^ Calcd for C_24_H_29_O_4_^+^ 381.2060; Found 381.2063. IR (film): 3443, 2080 (C≡C), 1742 (C=O), 1464 (Ar), and 1122 cm^−1^.

*5-Butyl-4-(hex-1-yn-1-yl)-1-methoxy-1,3-dihydrobenzo[c]thiophene 2,2-dioxide* (**11**)

To a stirred solution of thioether 3 (500 mg, 1.65 mmol) in CH_2_Cl_2_ (8 mL) at 0 °C was added *m*-CPBA (761 mg, 3.31 mmol). The resulting mixture was stirred at room temperature for 30 min before it was quenched by the addition of NaHCO_3_ (10 mL, satd aq). The layers were separated, and the aqueous layer was extracted with CH_2_Cl_2_ (3 × 10 mL). The combined organic layers were washed with brine (10 mL), dried (Na_2_SO_4_), and concentrated in vacuo. Flash column chromatography (silica gel, hexanes:EtOAc = 10:1) afforded sulfone 11 (425 mg, 77%) as a light yellow oil. ^1^H NMR (400 MHz, CDCl_3_): δ 7.28 (d, *J* = 8.0 Hz, 1H, Ar*H*), 7.23 (d, *J* = 8.0 Hz, 1H, Ar*H*), 5.26 (s, 1H, CH_3_OC*H*), 4.40 (d, *J* = 16.3 Hz, 1H, SO_2_C*H*), 4.33 (d, *J* = 16.3 Hz, 1H, SO_2_C*H*), 3.82 (s, 3H, OC*H*_3_), 2.80–2.73 (m, 2H, ArC*H*_2_), 2.47 (t, *J* = 6.9 Hz, 2H, C≡CC*H*_2_), 1.61–1.56 [m, 4H, (C*H*_2_CH_2_CH_3_)_2_], 1.51–1.46 (m, 2H, CH_2_C*H*_2_CH_3_), 1.38–1.32 (m, 2H, CH_2_C*H*_2_CH_3_), and 0.94 [dt, *J* = 11.0, 7.3 Hz, 6H, (CH_2_CH_2_C*H*_3_)_2_]. ^13^C NMR (101 MHz, CDCl_3_): δ 147.7, 133.5, 130.1, 129.5, 126.0, 121.8, 100.6, 96.3, 76.2, 59.9, 55.1, 34.6, 32.7, 30.9, 22.6, 22.1, 19.4, 14.0, and 13.7. HRMS (ESI) *m*/*z*: [M + H]^+^ Calcd for C_19_H_27_O_3_S^+^ 335.1675; Found 335.1675. IR (film): 3293, 2090 (C≡C), 1640, 1317, and 1208 cm^−1^.

*7-Butyl-8-(hex-1-yn-1-yl)-4-methoxy-2-methyl-3a,4,9,9a-tetrahydro-1H-cyclopenta[b]naphthalene-1,3(2H)-dione* (**14**)

To a stirred solution of sulfone 11 (150 mg, 0.45 mmol) in chlorobenzene (4 mL) was added *N*-methylmaleimide 5 (150 mg, 1.35 mmol). The resulting mixture was stirred at 100 °C for 6 h before it was quenched by the addition of NaHCO_3_ (10 mL, satd aq). The layers were separated, and the aqueous layer was extracted with CH_2_Cl_2_ (3 × 10 mL). The combined organic layers were washed with brine (10 mL), dried (Na_2_SO_4_), and concentrated in vacuo. Flash column chromatography (silica gel, hexanes:EtOAc = 5:1) afforded 14 (132 mg, 78%) as a light yellow oil. ^1^H NMR (400 MHz, CDCl_3_): δ 7.05 (d, *J* = 2.7 Hz, 2H, Ar*H*), 4.66 (d, *J* = 3.8 Hz, 1H, CH_3_OC*H*), 3.81 (dd, *J* = 14.9, 8.0 Hz, 1H, COC*H_a_*), 3.10 (m, 1H, Ar*H_a_*H_b_), 3.05 (s, 3H, OC*H*_3_), 3.03 (m, 1H, COC*H_b_*), 2.99 (s, 3H, CO_2_C*H*_3_), 2.93 (dd, *J* = 14.9, 10.1 Hz, 1H, ArH_a_*H_b_*), 2.77 (td, *J* = 7.5, 3.8 Hz, 2H, ArC*H*_2_), 2.52 (t, *J* = 7.0 Hz, 2H, C≡CC*H*_2_), 1.67–1.58 [m, 4H, (C*H*_2_CH_2_CH_3_)_2_], 1.56–1.49 (m, 2H, CH_2_C*H*_2_CH_3_), 1.44–1.33 [m, 2H, CH_2_C*H*_2_CH_3_), and 0.96 [dt, *J* = 10.7, 7.3 Hz, 6H, (CH_2_CH_2_C*H*_3_)_2_]. ^13^C NMR (101 MHz, CDCl3): δ 180.2, 177.2, 146.3, 138.5, 132.2, 127.5, 126.3, 123.4, 99.5, 77.8, 76.7, 56.0, 46.7, 38.3, 35.0, 32.9, 31.1, 25.5, 25.0, 22.9, 22.2, 19.6, 14.1, and 13.8. HRMS (ESI) *m*/*z*: [M + H]^+^ Calcd for C_24_H_32_NO_3_^+^ 382.2377; Found 382.2377. IR (neat): 3447 2115 (C≡C), 1704 (C=O), 1644, and 1434 (Ar) cm^−1^.

*7-Butyl-8-(hex-1-yn-1-yl)-4-methoxy-3a,4,9,9a-tetrahydronaphtho [2,3-c]furan-1,3-dione* (**15**)

To a stirred solution of sulfone 11 (140 mg, 0.42 mmol) in chlorobenzene (3 mL) was added maleic anhydride 12 (205 mg, 2.09 mmol). The resulting mixture was stirred at 100 °C for 5 h before it was quenched by the addition of NaHCO_3_ (10 mL, satd aq). The layers were separated, and the aqueous layer was extracted with CH_2_Cl_2_ (3 × 10 mL). The combined organic layers were washed with brine (10 mL), dried (Na_2_SO_4_), and concentrated in vacuo. Flash column chromatography (silica gel, hexanes:EtOAc = 10:1) afforded 15 (95 mg, 63%) as a bright yellow oil. ^1^H NMR (400 MHz, CDCl_3_): δ 7.07 (s, 2H, Ar*H*), 4.67 (d, *J* = 3.6 Hz, 1H, CH_3_OC*H*), 3.83 (dd, *J* = 15.5, 9.3 Hz, 1H, COC*H*_a_), 3.44 (dt, *J* = 10.7, 9.1 Hz, 1H, Ar*H_a_*H_b_)), 3.24 (dd, *J* = 10.7, 3.7 Hz, 1H, ArH_a_*H_b_*)), 3.11 (dd, *J* = 15.9, 7.0 Hz, 1H, ArH_a_*H_b_*)), 3.05 (s, 3H, OC*H*_3_), 2.78 (td, *J* = 7.7, 5.5 Hz, 2H, ArC*H*_2_), 2.52 (t, *J* = 7.0 Hz, 2H, C≡CC*H*_2_), 1.67–1.57 [m, 4H, (C*H*_2_CH_2_CH_3_)_2_], 1.55–1.49 (m, 2H, CH_2_C*H*_2_CH_3_), 1.43–1.35 (m, 2H, CH_2_C*H*_2_CH_3_), and 0.97 [dt, *J* = 10.9, 7.3 Hz, 6H, (CH_2_CH_2_C*H*_3_)_2_]. ^13^C NMR (101 MHz, CDCl_3_): δ 174.2, 171.3, 146.9, 137.1, 130.8, 127.7, 126.6, 123.6, 100.1, 77.5, 76.5, 56.1, 47.6, 38.1, 35.0, 32.8, 31.0, 25.0, 22.8, 22.2, 19.5, 14.1, and 13.8. HRMS (ESI) *m*/*z*: [M + H]^+^ Calcd for C_23_H_29_O_4_^+^ 369.2060; Found 369.2059. IR (film): 3443, 2079 (C≡C), 1641 (C=O), 1454 (Ar), and 1016 cm^−1^.

*6-Butyl-5-(hex-1-yn-1-yl)anthracene-1,4-dione* (**16**)

To a stirred solution of sulfone 11 (145 mg, 0.43 mmol) in chlorobenzene (3 mL) was added 1,4-benzoquinone 6 (141 mg, 1.30 mmol). The resulting mixture was stirred at 100 °C for 6 h before it was quenched by the addition of NaHCO_3_ (10 mL, satd aq). The layers were separated, and the aqueous layer was extracted with CH_2_Cl_2_ (3 × 10 mL). The combined organic layers were washed with brine (10 mL), dried (Na_2_SO_4_), and concentrated in vacuo. Flash column chromatography (silica gel, hexanes:EtOAc = 10:1) afforded 16 (100 mg, 66%) as a bright yellow oil. ^1^H NMR (400 MHz, CDCl_3_): δ 9.06 (s, 1H, COC*H*=CH), 8.51 (s, 1H, COCH=C*H*), 7.85 (d, *J* = 8.4 Hz, 1H, Ar*H*), 7.51 (d, *J* = 8.4 Hz, 1H, Ar*H*), 7.04 (q, *J* = 1.7 Hz, 2H, Ar*H*), 3.03–2.91 (m, 2H, ArC*H*_2_), 2.67 (t, *J* = 6.9 Hz, 2H, C≡CC*H*_2_), 1.77–1.66 [m, 4H, (C*H*_2_CH_2_CH_3_)_2_], 1.64–1.58 (m, 2H, CH_2_C*H*_2_CH_3_), 1.47–1.37 (m, 2H, CH_2_C*H*_2_CH_3_), and 1.00 [dt, *J* = 24.9, 7.7 Hz, 6H, (CH_2_CH_2_C*H*_3_)_2_]. ^13^C NMR (101 MHz, CDCl3): δ 184.9 (2C), 147.6, 140.3, 140.0, 135.6, 133.3, 131.1, 129.2, 128.9, 128.7, 128.0, 127.6, 123.0, 102.0, 76.2, 35.3, 32.8, 31.0, 22.8, 22.3, 19.8, 14.1, and 13.8. HRMS (ESI) *m*/*z*: [M + H]^+^ Calcd for C_24_H_25_O_2_^+^ 345.1849; Found 345.1845. IR (film): 2957, 2089 (C≡C), 1667 (C=O), 1457 (Ar), and 1056 cm^−1^.

*4-Butyl-3-(hex-1-yn-1-yl)-2-methylbenzaldehyde* (**17**)

To a stirred solution of sulfone 11 (115 mg, 0.34 mmol) in chlorobenzene (3 mL) was added dimethyl fumarate 13 (145 mg, 1.0 mmol). The resulting mixture was stirred at 100 °C for 4 h before it was quenched by the addition of NaHCO_3_ (10 mL, satd aq). The layers were separated, and the aqueous layer was extracted with CH_2_Cl_2_ (3 × 10 mL). The combined organic layers were washed with brine (10 mL), dried (Na_2_SO_4_), and concentrated in vacuo. Flash column chromatography (silica gel, hexanes:EtOAc = 20:1) afforded 17 (80 mg, 49%) as a colorless oil. ^1^H NMR (400 MHz, CDCl_3_): δ 10.25 (s, 1H, C*H*O), 7.64 (d, *J* = 7.9 Hz, 1H, Ar*H*), 7.17 (d, *J* = 7.9 Hz, 1H, Ar*H*), 2.86–2.80 (m, 2H, ArC*H*_2_), 2.78 (s, 3H, ArC*H*_3_), 2.53 (t, *J* = 6.9 Hz, 2H, C≡CC*H*_2_), 1.69–1.59 [m, 4H, (C*H*_2_CH_2_CH_3_)_2_], 1.55–1.48 (m, 2H, CH_2_C*H*_2_CH_3_), 1.43–1.36 (m, 2H, CH_2_C*H*_2_CH_3_), and 0.96 [q, *J* = 7.6 Hz, 6H, (CH_2_CH_2_C*H*_3_)_2_]. ^13^C NMR (101 MHz, CDCl_3_): δ 192.4, 151.5, 143.0, 132.2, 130.2, 126.5, 125.7, 100.3, 77.0, 35.5, 32.4, 31.0, 22.8, 22.2, 19.5, 16.9, 14.1, and 13.8. HRMS (ESI) *m*/*z*: [M + H]^+^ Calcd for C_18_H_25_O^+^ 257.1900; Found 257.1903. IR (film): 2827, 2098 (C≡C), 1715 (C=O), 1455 (Ar), and 1054 cm^−1^.

*Dimethyl (2R,3S)-6-butyl-5-(hex-1-yn-1-yl)-1-methoxy-1,2,3,4-tetrahydronaphthalene-2,3-dicarboxylate* (**18**)

To a stirred solution of sulfone 11 (85 mg, 0.25 mmol) in chlorobenzene (2 mL) were added dimethyl fumarate 13 (108 mg, 0.75 mmol) and ZnO (61 mg, 0.75 mmol). The resulting mixture was stirred at 100 °C for 6 h before it was quenched by the addition of NaHCO_3_ (10 mL, satd aq). The layers were separated, and the aqueous layer was extracted with CH_2_Cl_2_ (3 × 10 mL). The combined organic layers were washed with brine (10 mL), dried (Na_2_SO_4_), and concentrated in vacuo. Flash column chromatography (silica gel, hexanes:EtOAc = 10:1) afforded 18 (80 mg, 75%) as a light yellow solid. ^1^H NMR (400 MHz, CDCl_3_): δ 7.22 (d, *J* = 8.0 Hz, 0.35H, Ar*H*, minor), 7.07 (t, *J* = 7.7 Hz, 1.25H, Ar*H*, major), 7.04 (d, *J* = 8.0 Hz, 0.35H, Ar*H*, minor), 4.78 (d, *J* = 8.0 Hz, 0.35H, CH_3_OC*H*, minor), 4.56 (t, *J* = 2.6 Hz, 0.63H, CH_3_OC*H*, major), 3.77 (s, 3.8H, CO_2_C*H*_3_, major), 3.75 (s, 1H, CO_2_C*H*_3_, minor), 3,73 (s, 1H, CO_2_C*H*_3_, minor), 3.51–3.33 (m, 2H, ArC*H*_2_CH), 3.3 (s, 1H, ArC*H*_2_CH minor), 3.26 (s, 1.9H, ArCH_2_C*H*, major), 3.18–2.76 (m, 2H, ArC*H*_2_C*H*), 2.74 (m, 2H, ArC*H*_2_), 2.48 (m, 2H, C≡CC*H*_2_), 11.66–1.12 [m, 8H, (C*H*_2_C*H*_2_CH_3_)_2_], and 0.94 [m, 6H, CH_2_CH_2_C*H*_3_)_2_]. ^13^C NMR (101 MHz, CDCl_3_): δ 176.4, 174.05, 174.03 172.7, 145.9, 145.0, 136.7, 136.5, 132.2, 130.5, 128.8, 126.9, 126.3, 126.0, 123.5, 122.8, 100.6, 99.9, 79.0, 77.7, 57.0, 55.2, 52.4, 52.3, 52.2, 47.3, 46.4, 41.4, 36.9, 34.7, 34.6, 32.8, 32.8, 31.4, 31.1, 31.0, 30.0, 29.8, 22.8, 22.09, 22.07, 19.5, 14.1, 13.75, and 13.73. HRMS (ESI) *m*/*z*: [M + H]^+^ Calcd for C_25_H_35_O_5_^+^ 415.2479; Found 415.2475. IR (film): 2850, 2085 (C≡C), 1732 (C=O), 1483 (Ar), and 1030 cm^−1^.

## 4. Conclusions

In conclusion, we have developed a novel linker transformation strategy for PDDA cyclization. The results demonstrated that under basic conditions, thioether-tethered tetrayne undergoes cyclization in methanol solution to afford 1-methoxy thioether, which serves as an excellent diene. Additionally, upon oxidation, 1-methoxy thioethers are converted into sulfone compounds, exhibiting even greater potential as Diels–Alder reaction precursors. This innovative approach effectively transforms the linker, significantly expanding the applicability of PDDA reactions, particularly in the synthesis of fused polycyclic aromatic compounds.

## Figures and Tables

**Table 1:** Screening reaction conditions.

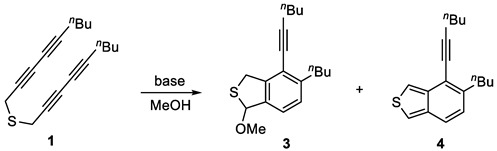					
Entry ^a^	Base	Equivalent	t (°C)	Ratio/3:4	Yield ^b^ of (3+4)(%)
1	K_2_CO_3_	0.5	50	--	--
2	K_2_CO_3_	1	50	4.2:1	51
3	K_2_CO_3_	5	50	3.0:1	61
4	Cs_2_CO_3_	0.5	50	4.3:1	57
5	Cs_2_CO_3_	1	50	3.4:1	61
6	Cs_2_CO_3_	5	50	1:1.8	70
7	Cs_2_CO_3_	1	rt	3.4:1	76
8	KOH	1	rt	2.3:1	55
9	*t*-BuOK	1	rt	3.0:1	65
10	Et_3_N	2	100	1.4:1	46
11	Et_3_N	5	100	1.8:1	58
12	TMP	1	70	2.5:1	58
13	TMP	10	70	3.2:1	75
14	DBU	0.5	rt	3.0:1	60
15	DBU	1	rt	2.7:1	67

^a^ Reaction conditions: tetrayne **1** (1 mmol), 0.2 M, 4–24 h. ^b^ isolated yields.

## Data Availability

All spectroscopic data obtained during this research are included in the Supplementary Materials (see above).

## Supplementary Materials

The following supporting information can be downloaded at: https://www.mdpi.com/article/10.3390/molecules30071617/s1, Figures S1–S27: ^1^H NMR spectrum and ^13^C NMR spectrum of compound **1**–**4**, **8a**, **8b**, **9**–**11**, **14**–**18**.
