# Supplementary material for: Utilizing the Pentadehydro-Diels–Alder Reaction for Polycyclic Aromatic Compound Synthesis: Diels–Alder-Based Linker Transformation
Source: Molecules. 2025 Apr 4;30(7):1617. doi: 10.3390/molecules30071617 (PMC11990327; doi:10.3390/molecules30071617)
Supplement: Supplementary file 1 [file molecules-30-01617-s001.zip › molecules-3563279-supplementary.pdf]

molecules

Supporting information for

Utilizing the Pentadehydro-Diels-Alder Reaction for Polycyclic Aromatic Compound Synthesis: Diels-Alder-Based Linker Transformation

Ying Xia , Qiaofeng Liang, Chenlong Zhu* and Bingfeng Sun*

School of Pharmaceutical Sciences, Nanjing Tech University, 30 South Puzhu Road, Nanjing 211816, China
* Correspondence: chenlongzhu@njtech.edu.cn
* Correspondence: bfsun@njtech.edu.cn

Table of Contents

Figure S1……………………………..…^{1}H NMR spectrum of **1**……………………………………...2
Figure S2…………………………….....^{13}C NMR spectrum of **1**………………………………………2
Figure S3……………………………..…^{1}H NMR spectrum of **2**……………………………………...3
Figure S4……………………………....^{13}C NMR spectrum of **2**…………………………..…………..3
Figure S5……………………………..…^{1}H NMR spectrum of **3**……………………………………...4
Figure S6……………………………....^{13}C NMR spectrum of **3**………………………………………4
Figure S7……………………………..…^{1}H NMR spectrum of **4**……………………………………...5
Figure S8……………………………..…^{1}H NMR spectrum of **8a**…………………………..……...6
Figure S9……………………………..…^{13}C NMR spectrum of **8a**……………………………………....6
Figure S10……………………………....^{1}H NMR spectrum of **8b**……………………………………....7
Figure S11……………………………....^{13}C NMR spectrum of **8b**……………………………………....7
Figure S12……………………………..…^{1}H NMR spectrum of **9**……………………………………..8
Figure S13……………………………....^{13}C NMR spectrum of **9**……………………………………...8
Figure S14……………………………..…^{1}H NMR spectrum of **10**……………………………..………...9
Figure S15……………………………....^{13}C NMR spectrum of **10**…………………….………..….....9
Figure S16……………………………..…^{1}H NMR spectrum of **11**……………………………………10
Figure S17……………………………....^{13}C NMR spectrum of **11**……………………………..………...10
Figure S18……………………………..…^{1}H NMR spectrum of **14**……………………………………..11
Figure S19……………………………....^{13}C NMR spectrum of **14**……………………………..………....11
Figure S20……………………………..…^{1}H NMR spectrum of **15**……………………………..…………12
Figure S21……………………………....^{13}C NMR spectrum of **15**……………………………..………....12
Figure S22……………………………..…^{1}H NMR spectrum of **16**……………………………………..13
Figure S23……………………………....^{13}C NMR spectrum of **16**…………………………..…………....13
Figure S24……………………………..…1H NMR spectrum of **17**……………………………………..14
Figure S25……………………………....^{13}C NMR spectrum of **17**……………………………….……...14
Figure S26……………………………..…^{1}H NMR spectrum of **18**……………………………………..15
Figure S27……………………………....^{13}C NMR spectrum of **18**…………………………..………..…..15

Figure S1 ^{1}H NMR spectrum of compound **1**

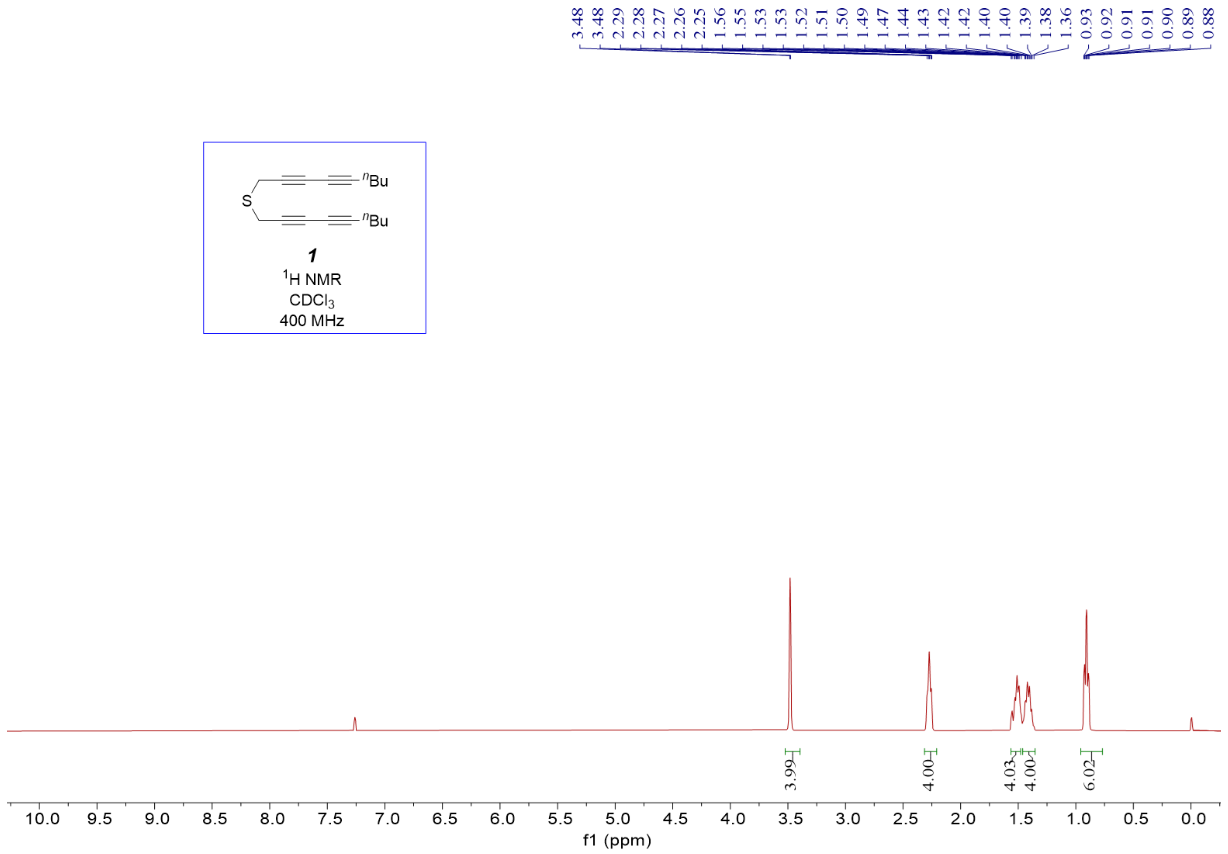

Figure S1 ^{1}H NMR Spectrum of Compound **1**

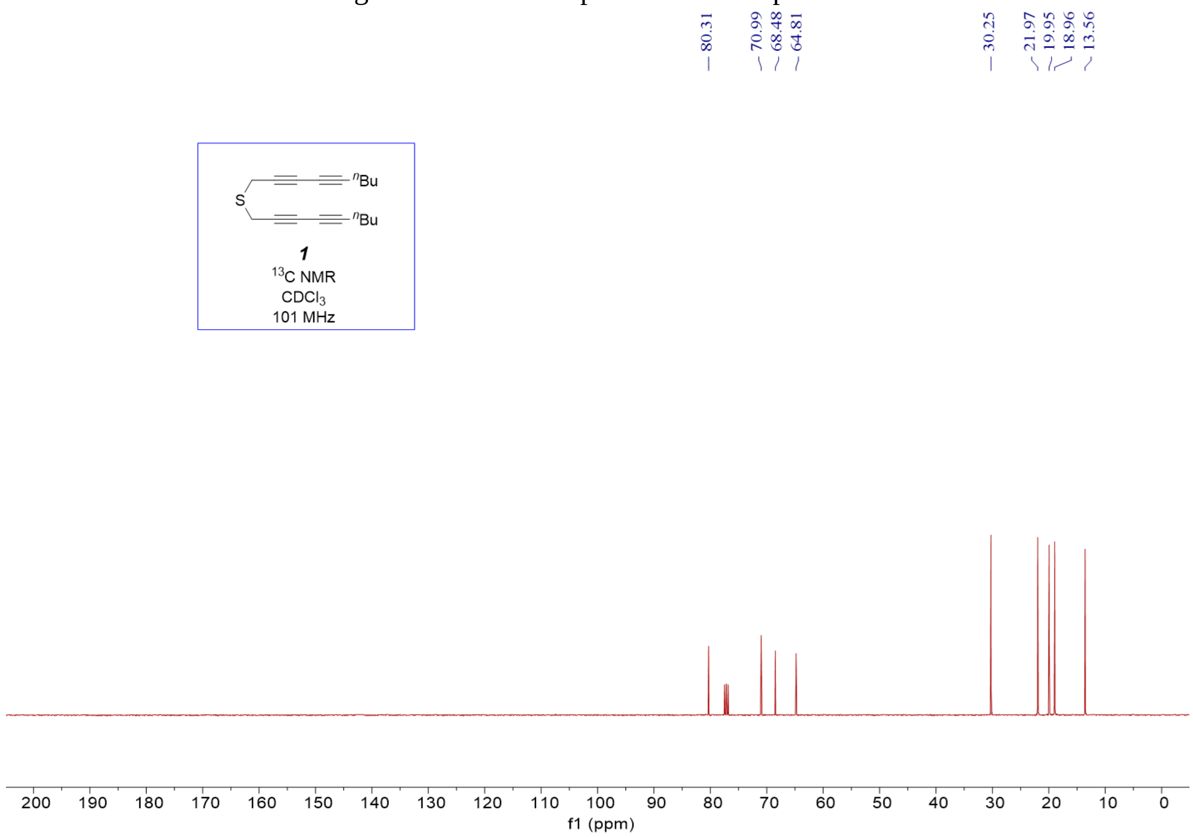

Figure S2 ^{13}C NMR Spectrum of Compound **1**

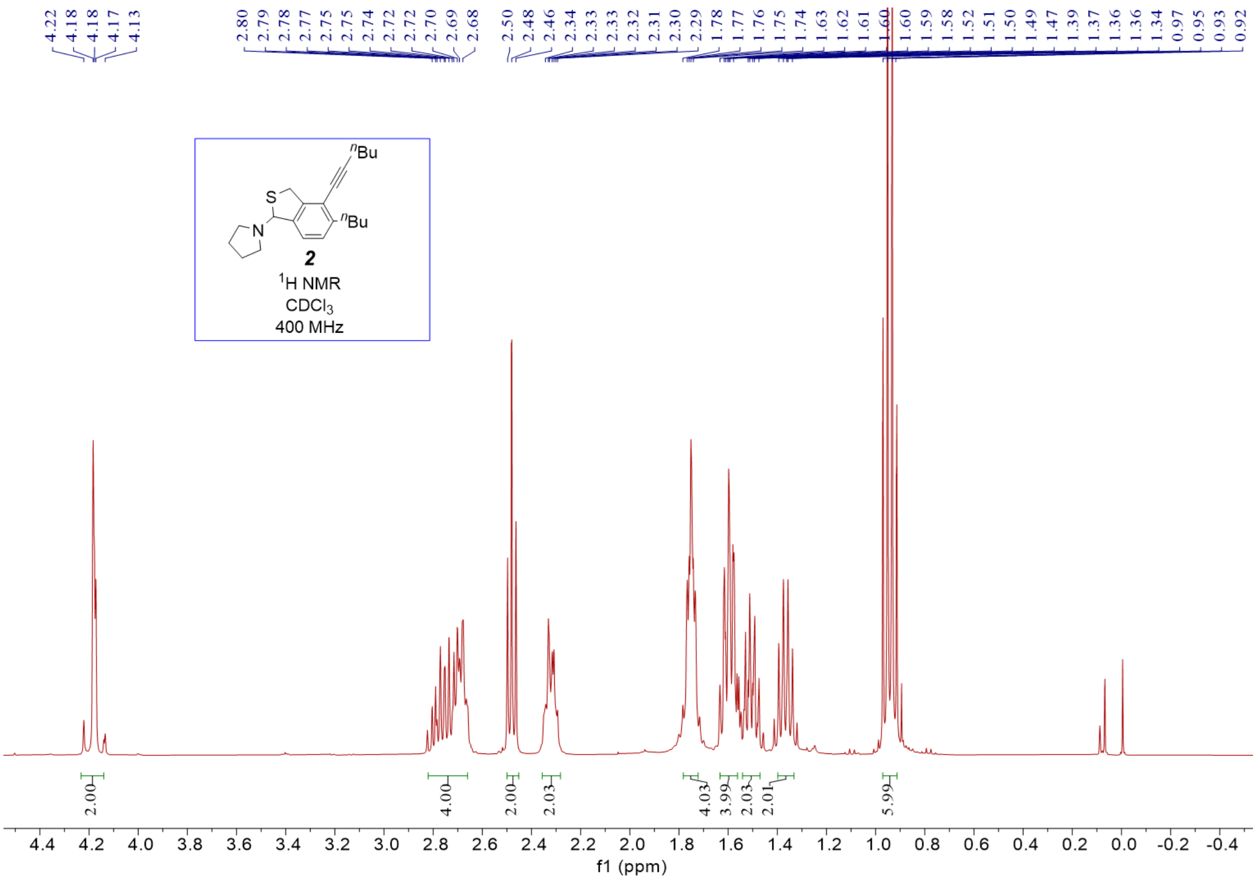

Figure S3 ^{1}H NMR Spectrum of Compound **2**

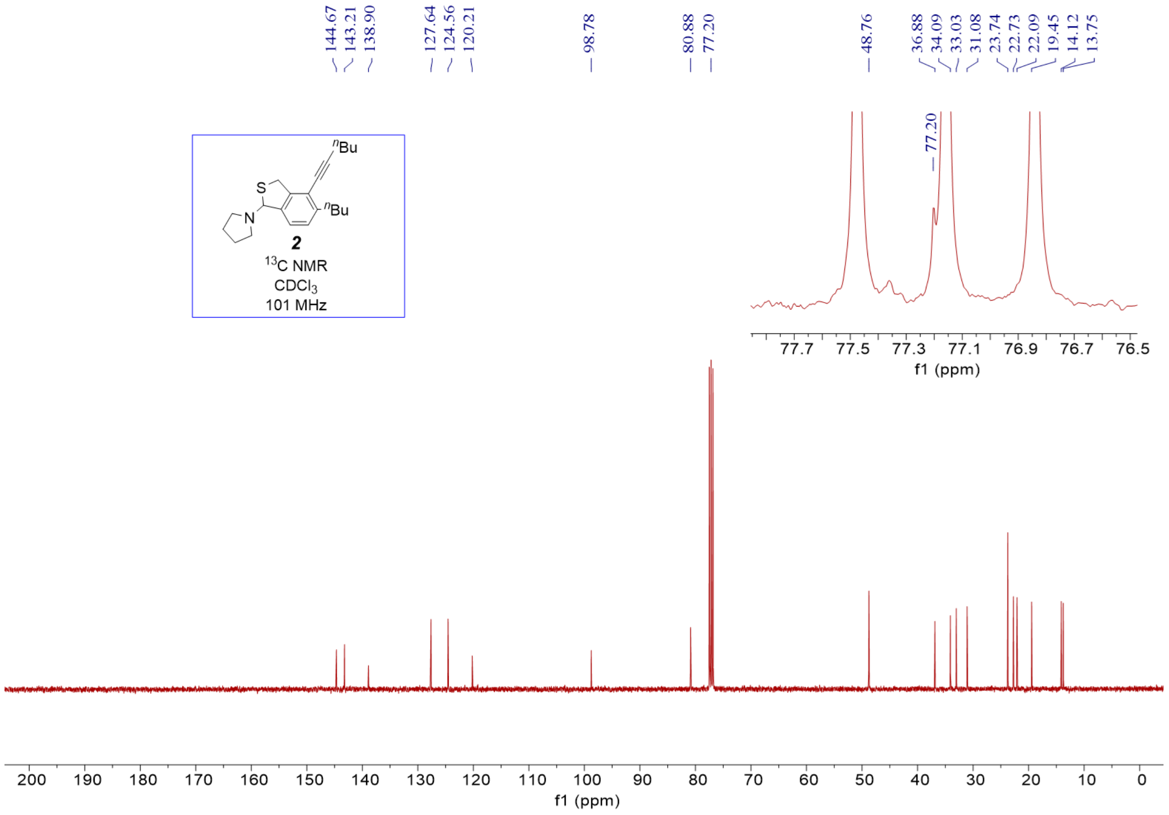

Figure S4 ^{13}C NMR Spectrum of Compound **2**

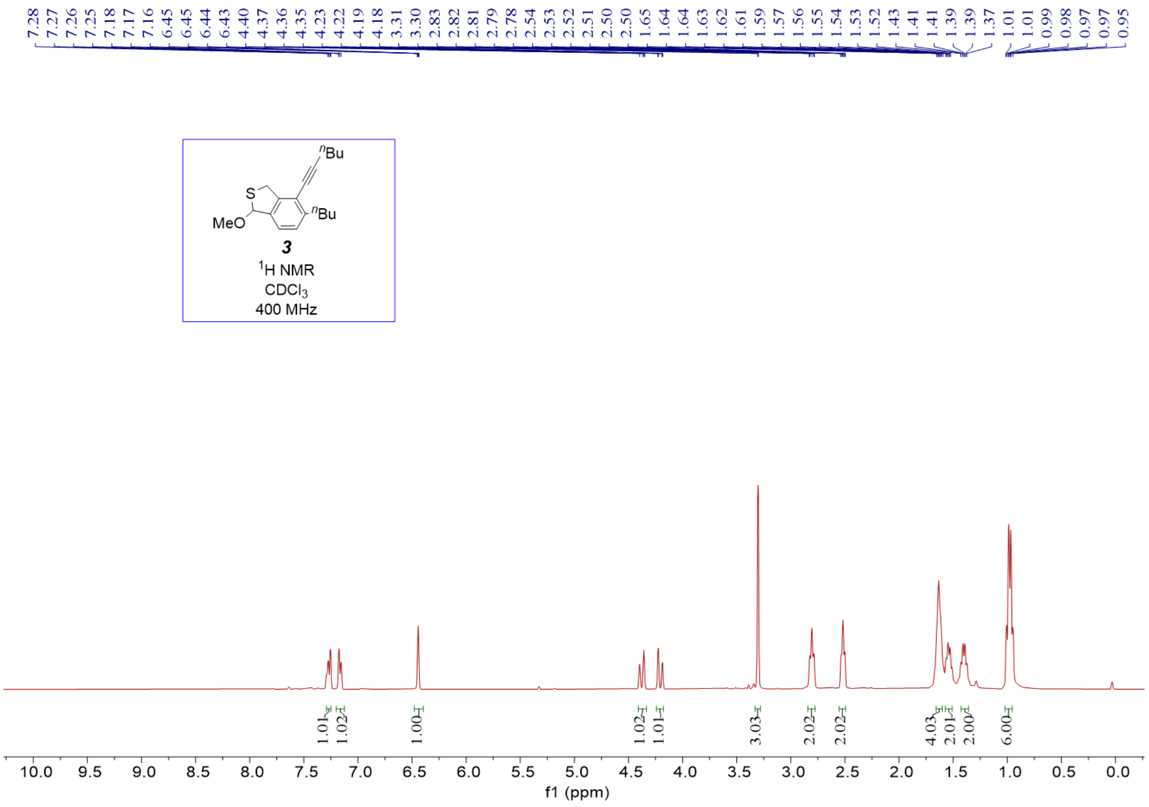

Figure S5 ^{1}H NMR Spectrum of Compound **3**

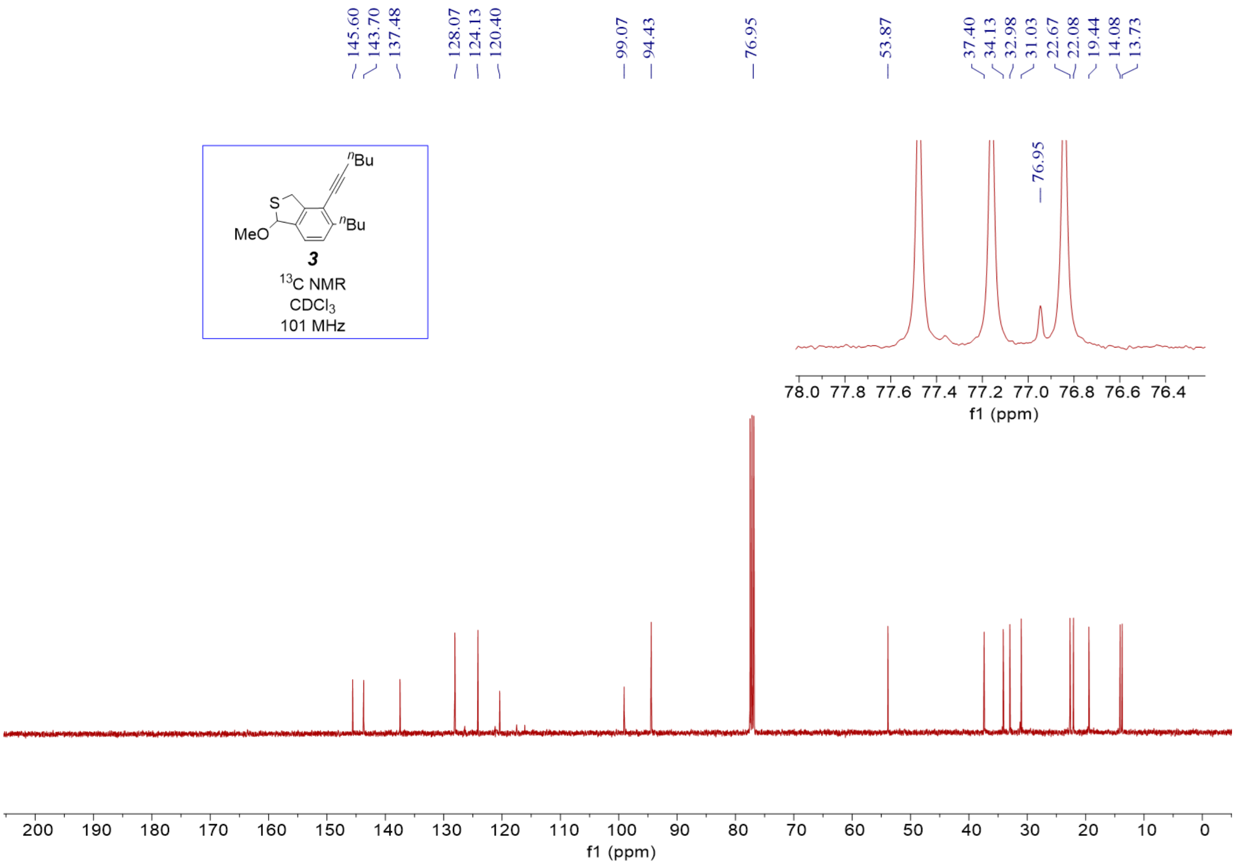

Figure S6 ^{13}C NMR Spectrum of Compound **3**

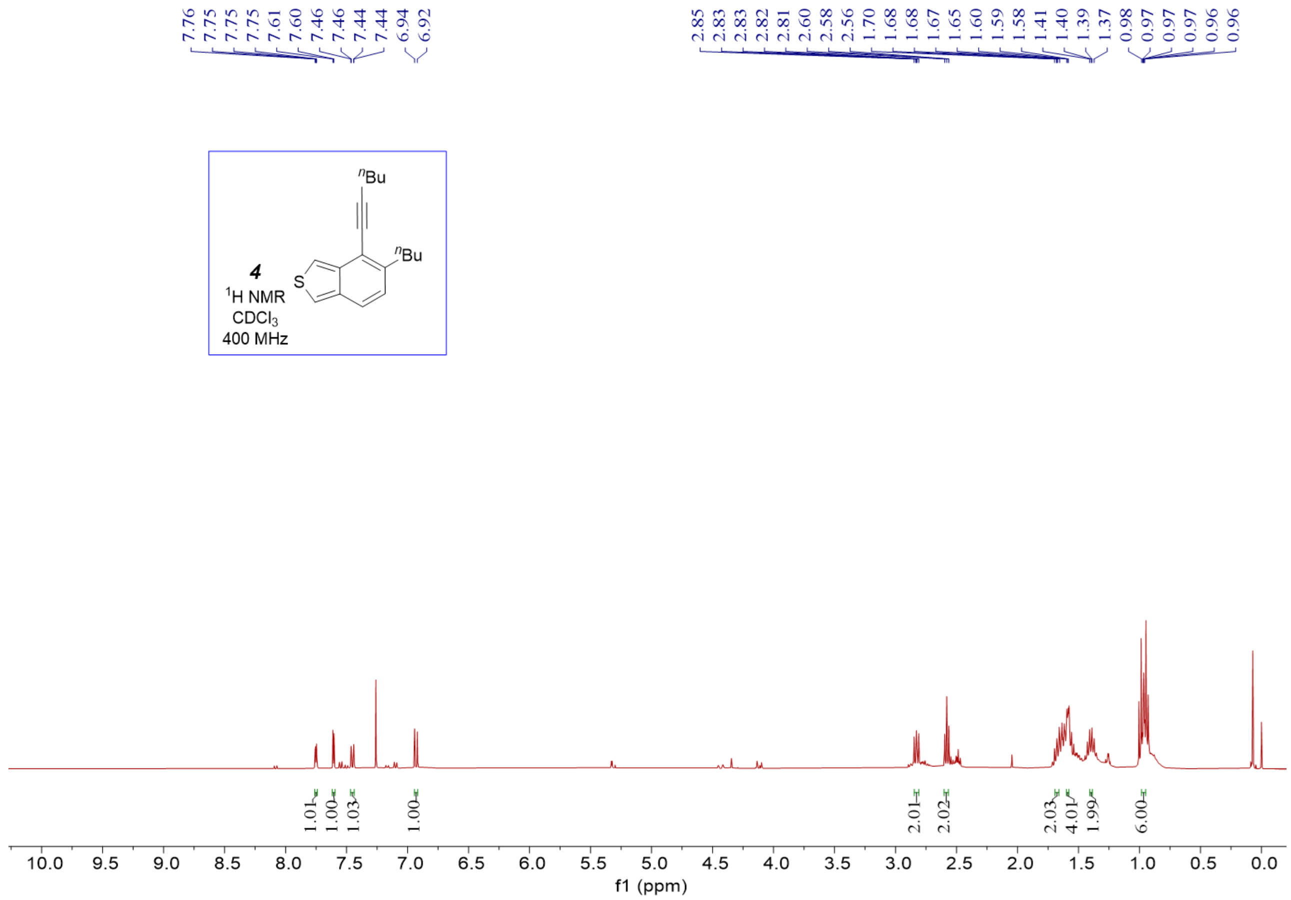

Figure S7 ^{1}H NMR Spectrum of Compound **4**

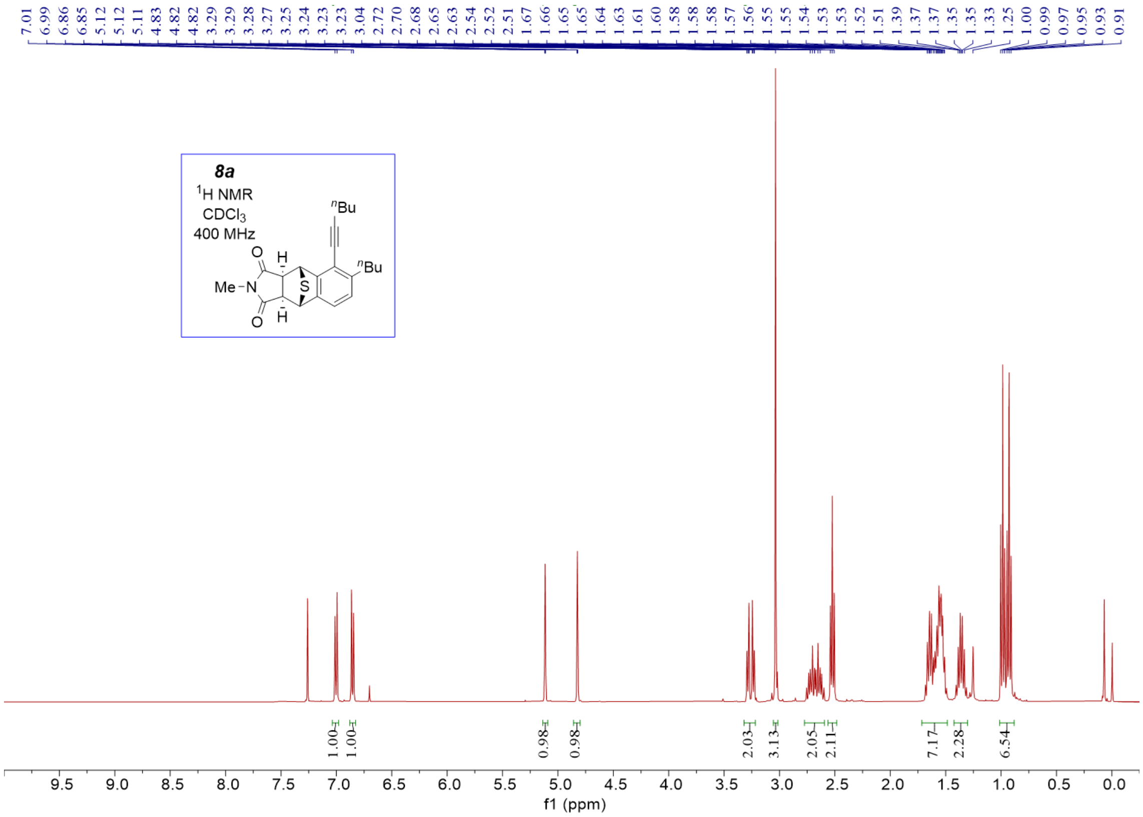

Figure S8 ^{1}H NMR Spectrum of Compound **8a**

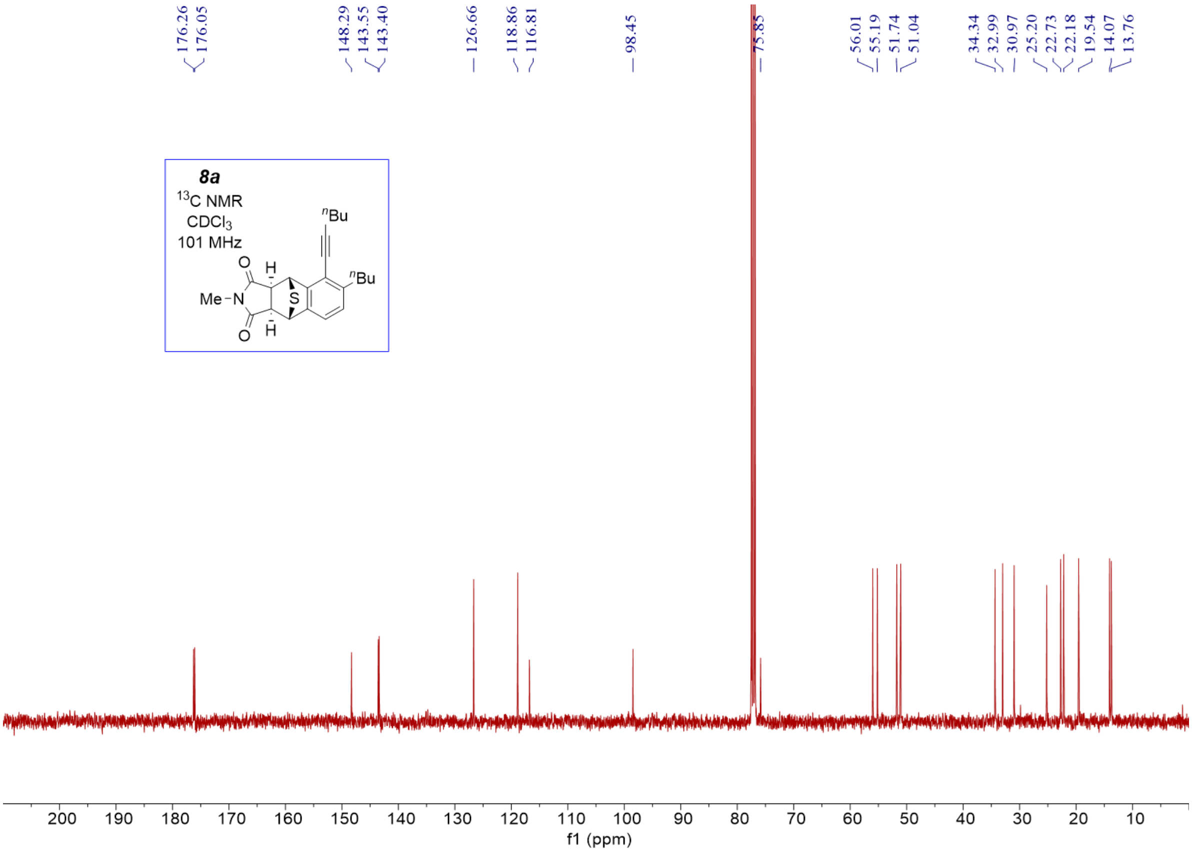

Figure S9 ^{13}C NMR Spectrum of Compound **8a**

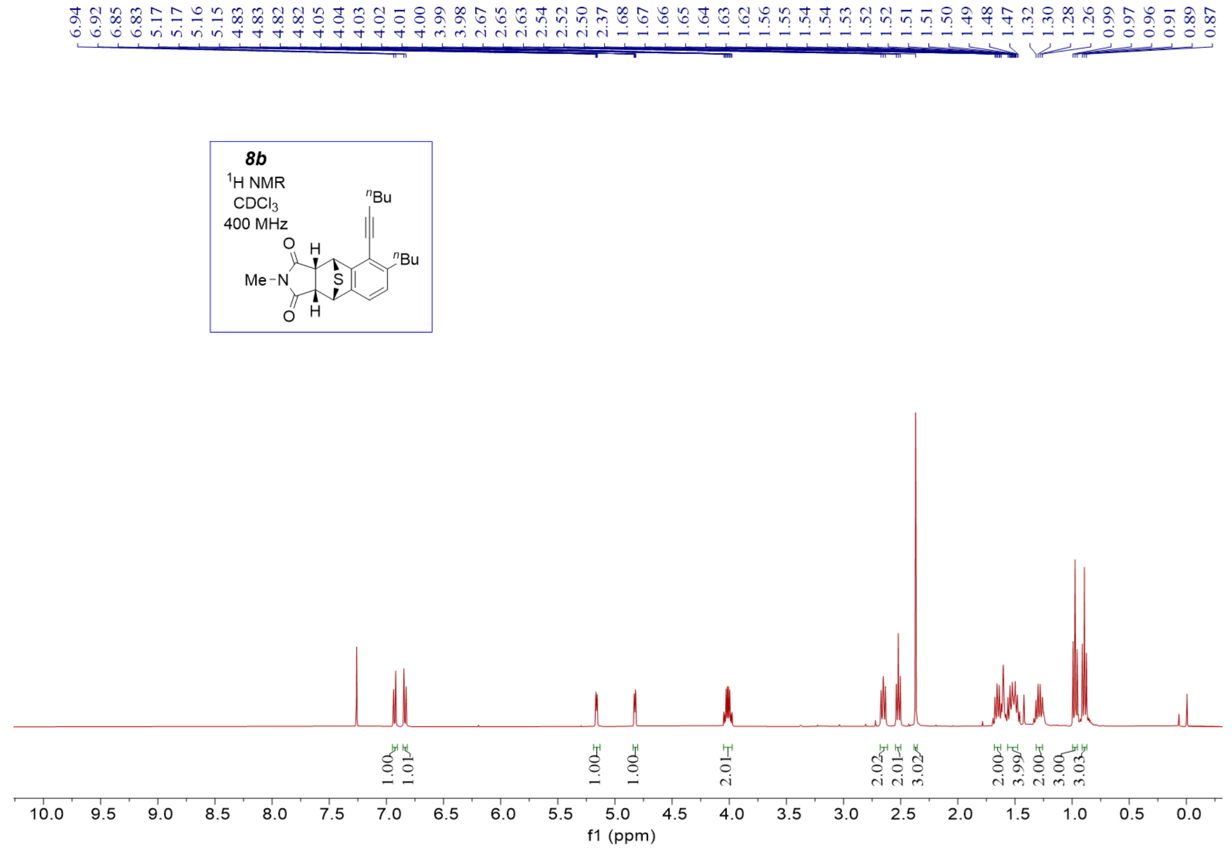

Figure S10 ^{1}H NMR Spectrum of Compound **8b**

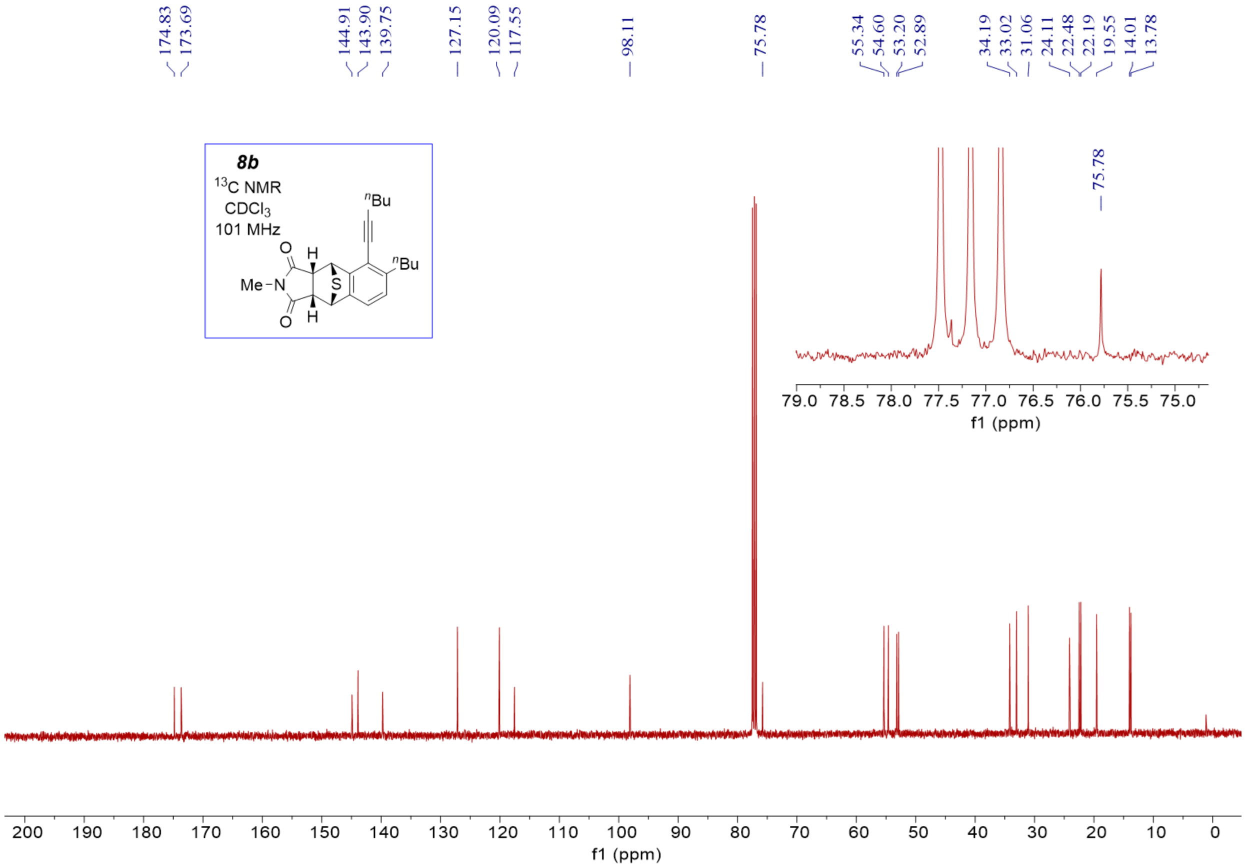

Figure S11 ^{13}C NMR Spectrum of Compound **8b**

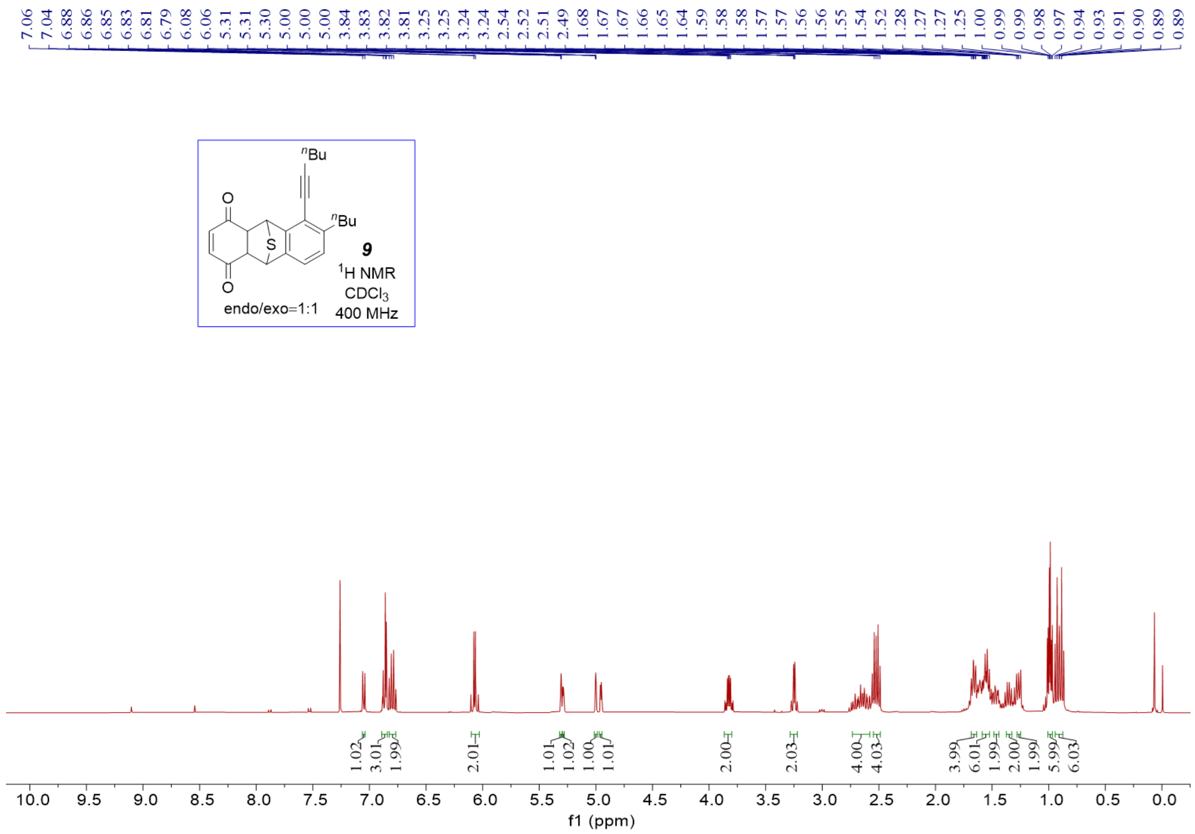

Figure S12 ^{1}H NMR Spectrum of Compound **9**

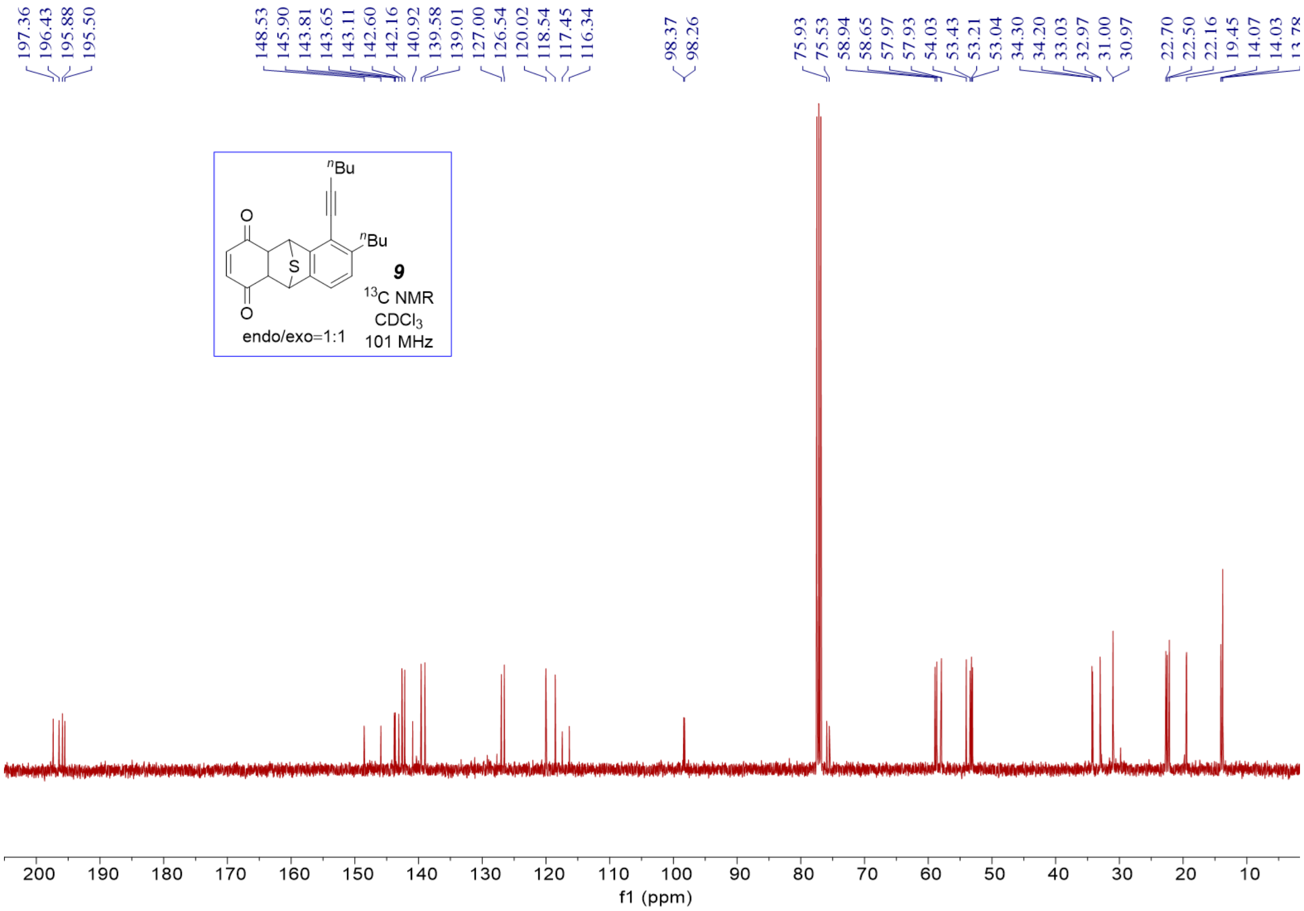

Figure S13 ^{13}C NMR Spectrum of Compound **9**

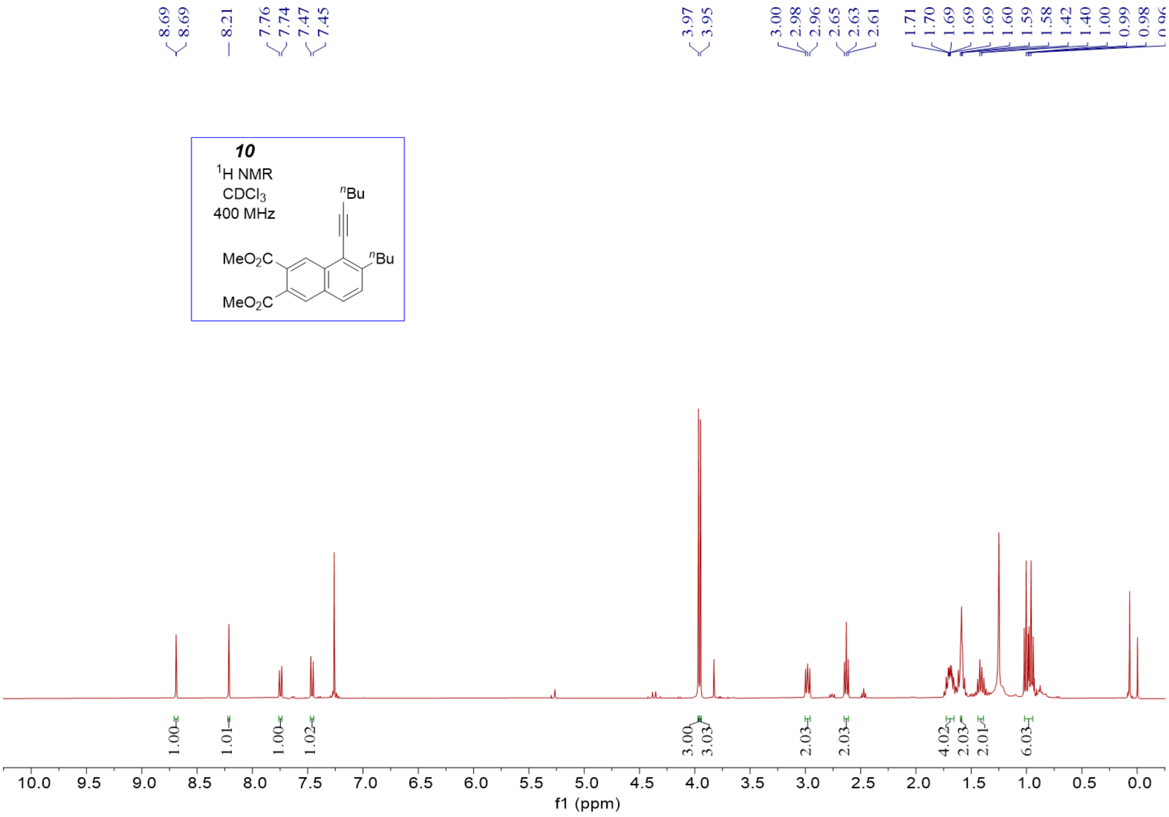

Figure S14 ^{1}H NMR Spectrum of Compound **10**

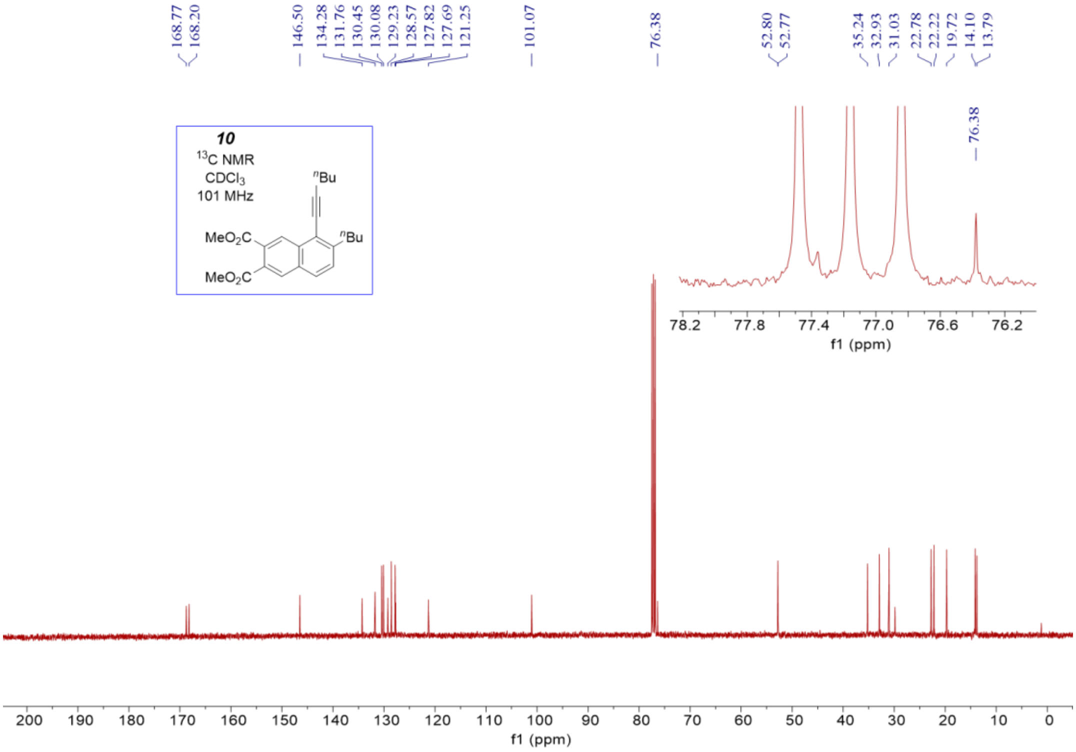

Figure S15 ^{13}C NMR Spectrum of Compound **10**

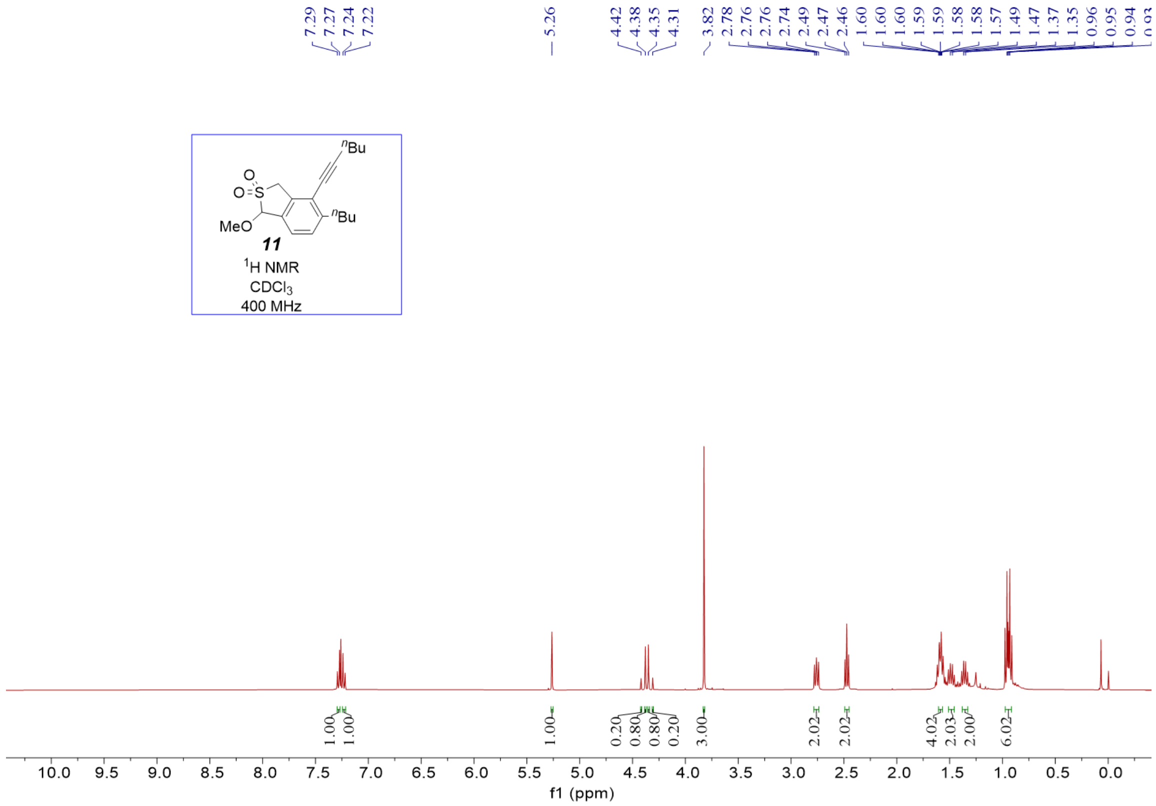

Figure S16 ^{1}H NMR Spectrum of Compound **11**

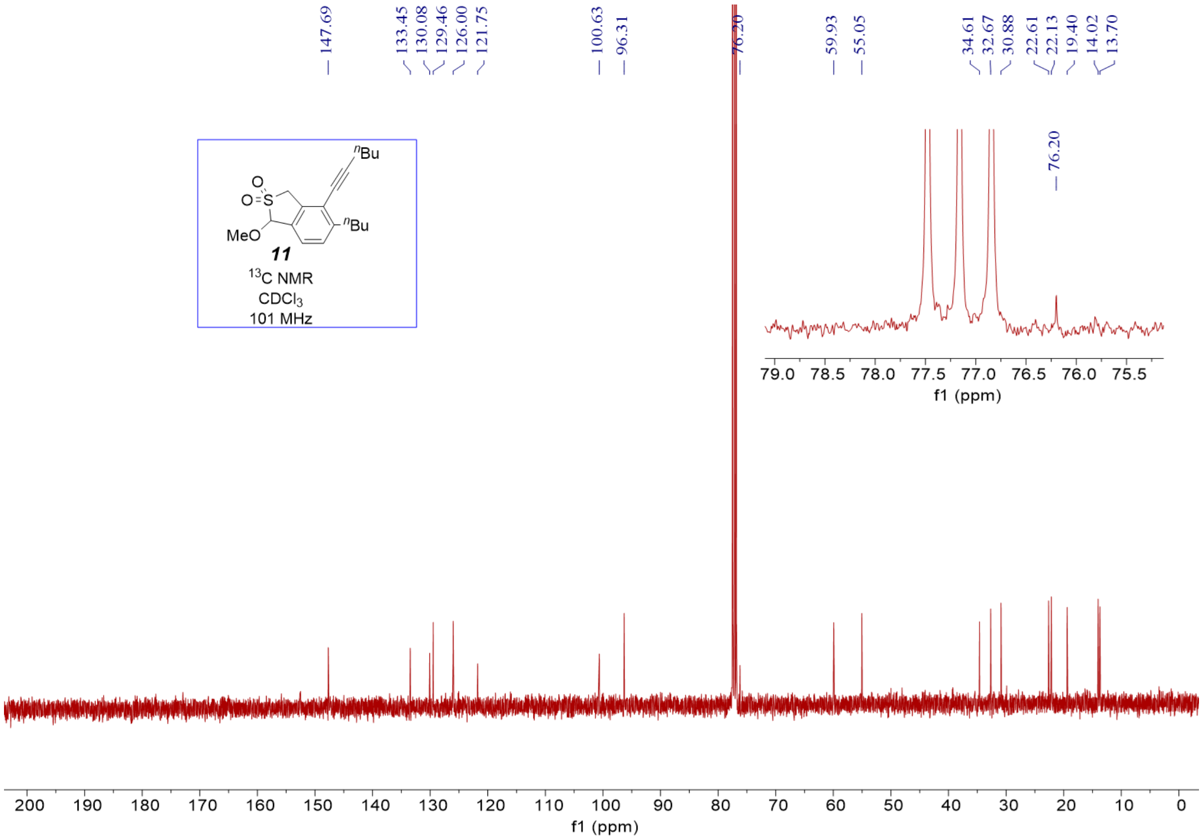

Figure S17 ^{13}C NMR Spectrum of Compound **11**

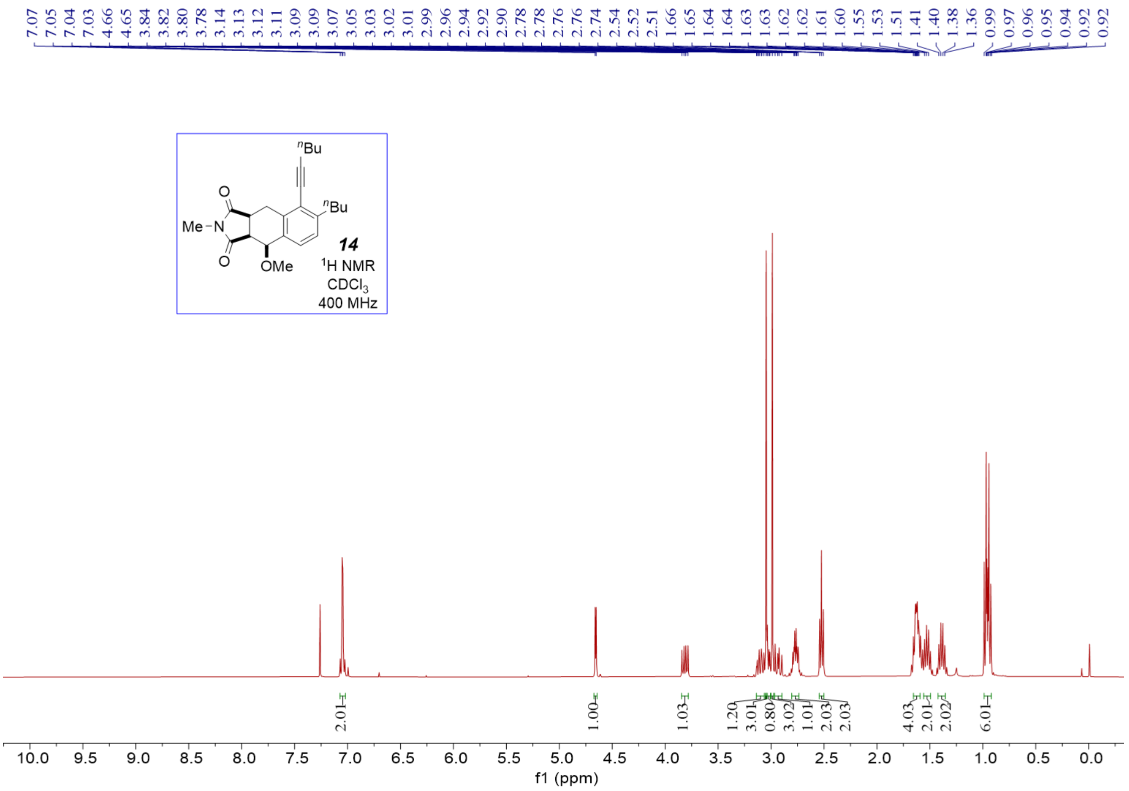

Figure S18 ^{1}H NMR Spectrum of Compound **14**

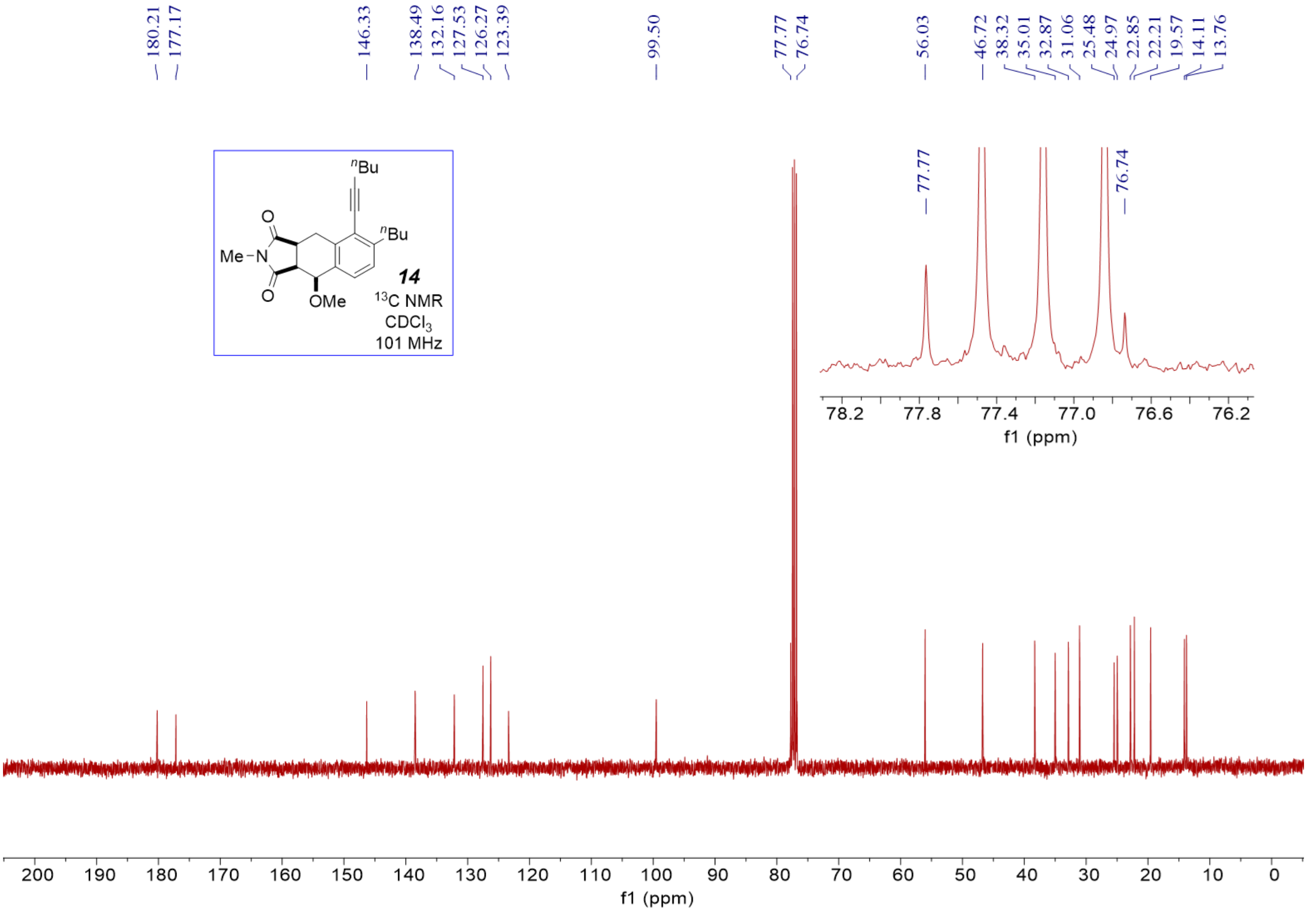

Figure S19 ^{13}C NMR Spectrum of Compound **14**

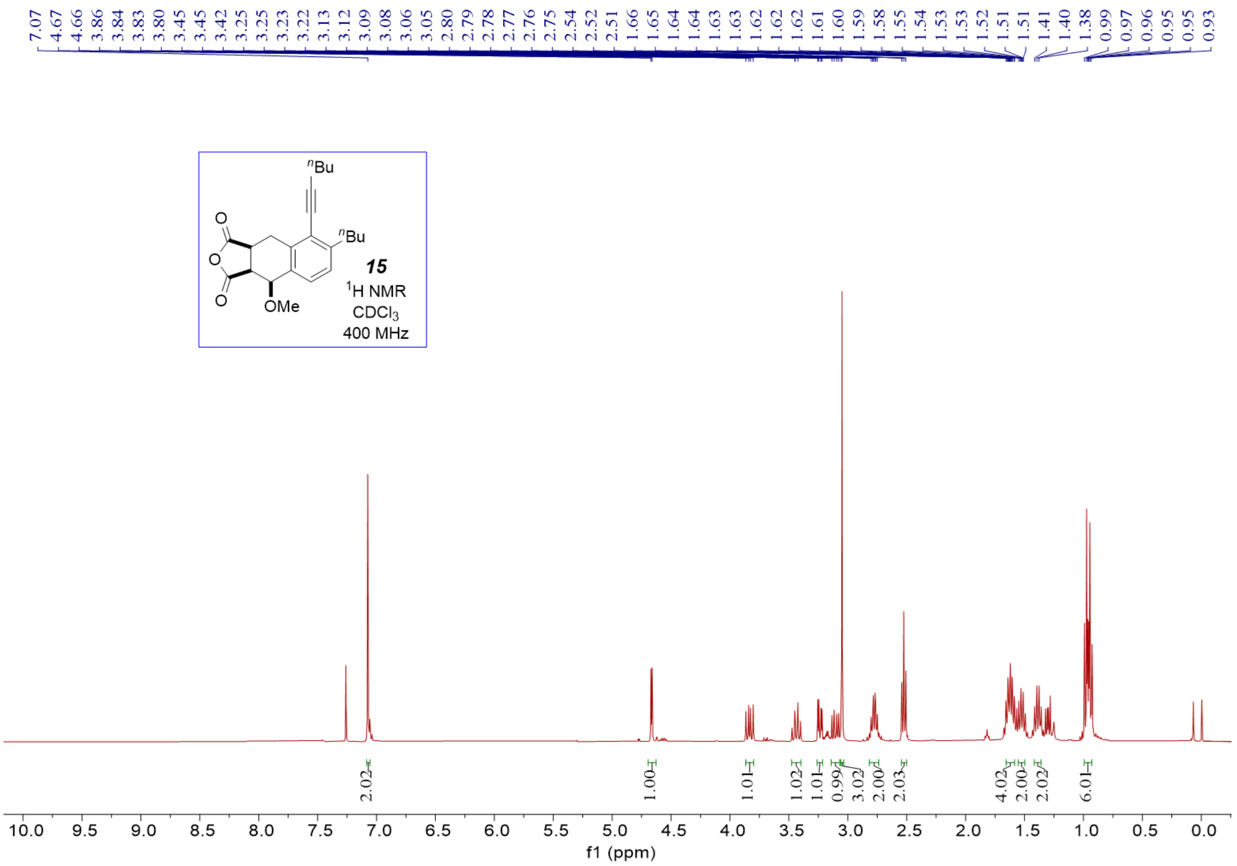

Figure S20 ^{1}H NMR Spectrum of Compound **15**

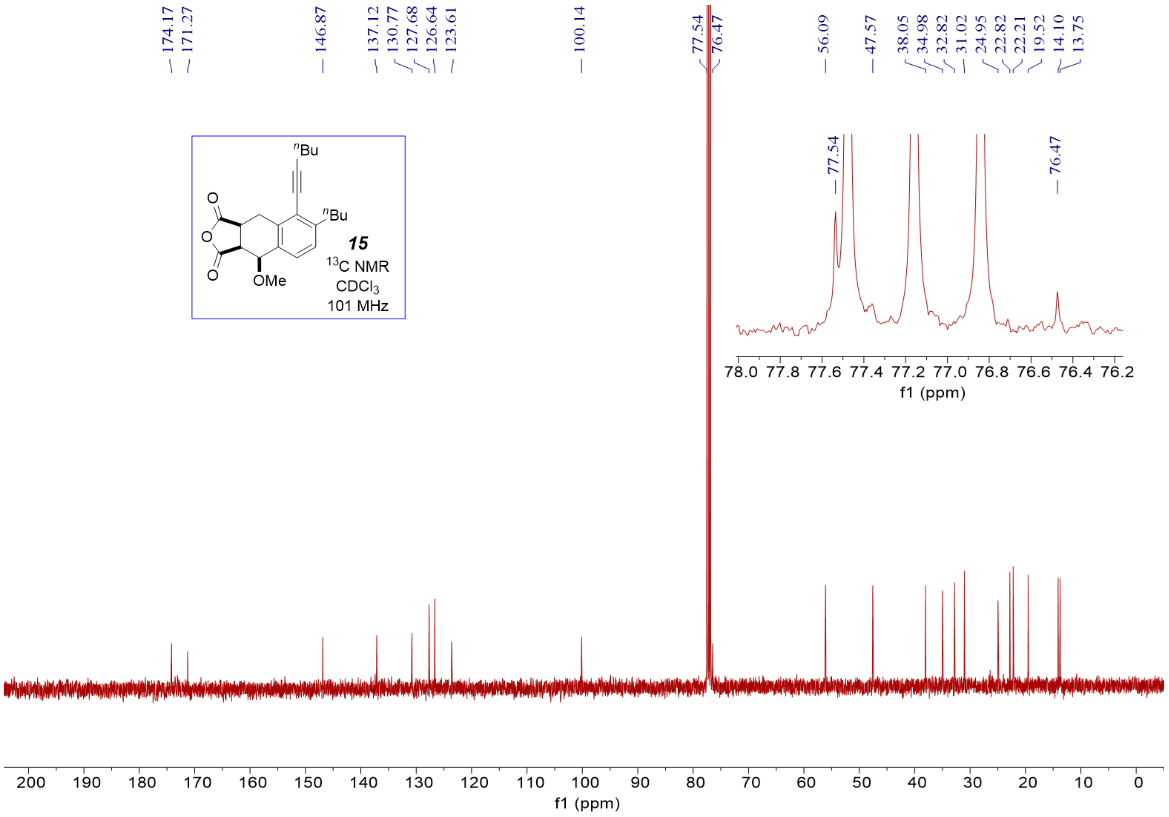

Figure S21 ^{13}C NMR Spectrum of Compound **15**

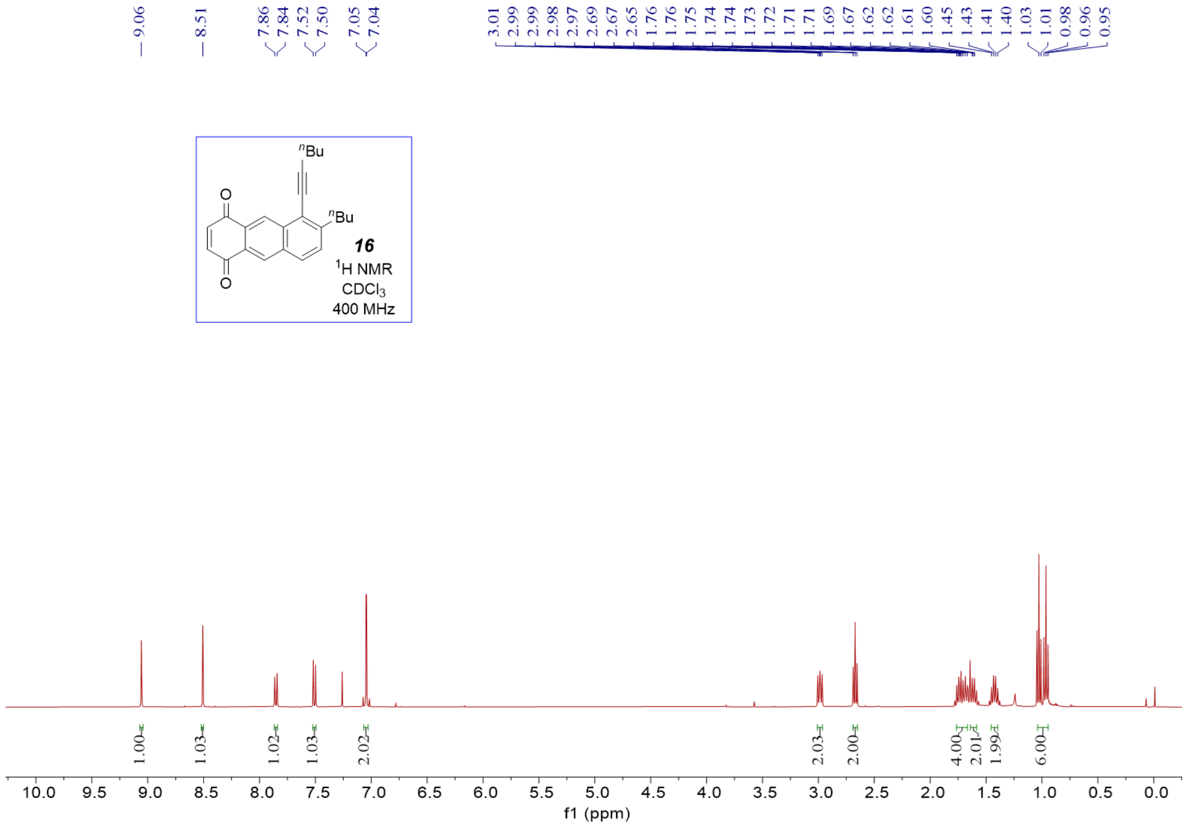

Figure S22 ^{1}H NMR Spectrum of Compound **16**

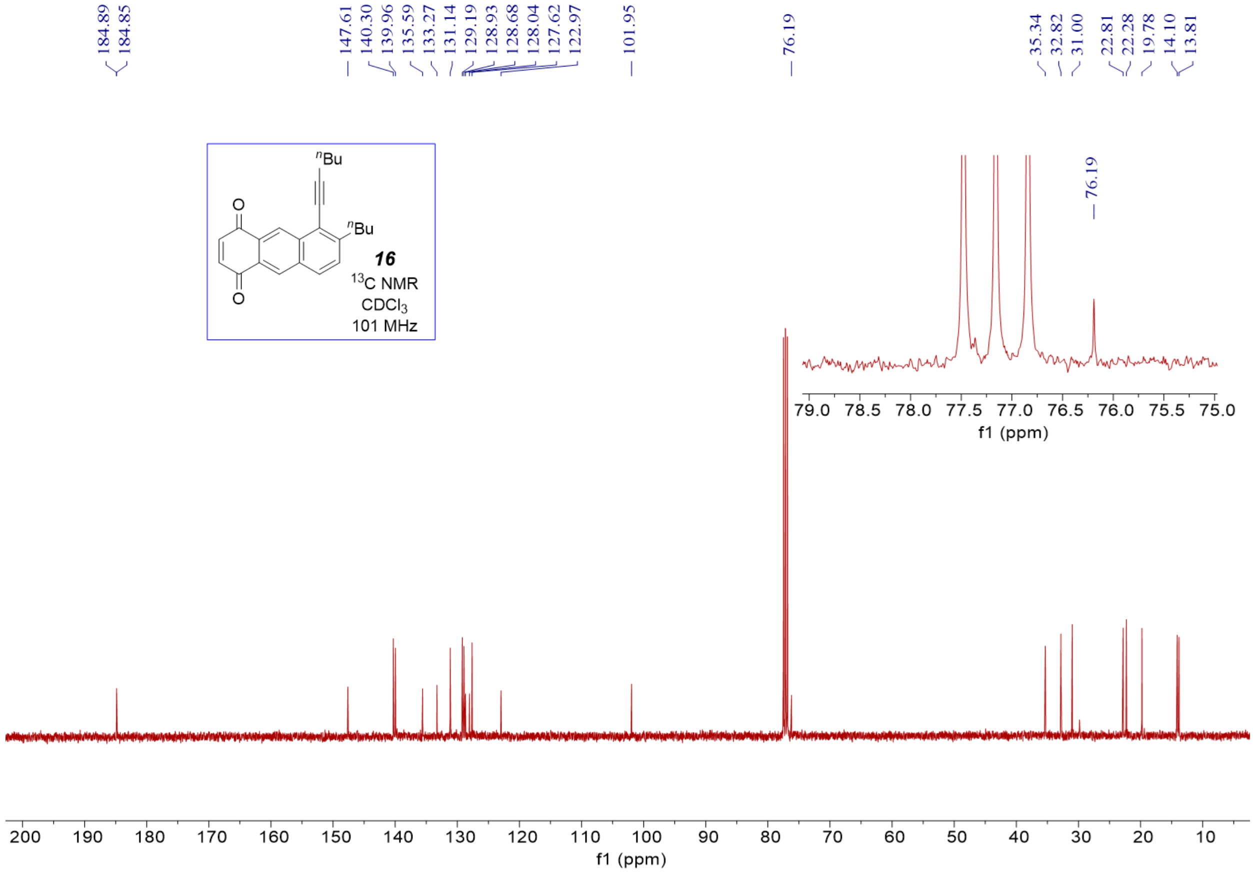

Figure S23 ^{13}C NMR Spectrum of Compound **16**

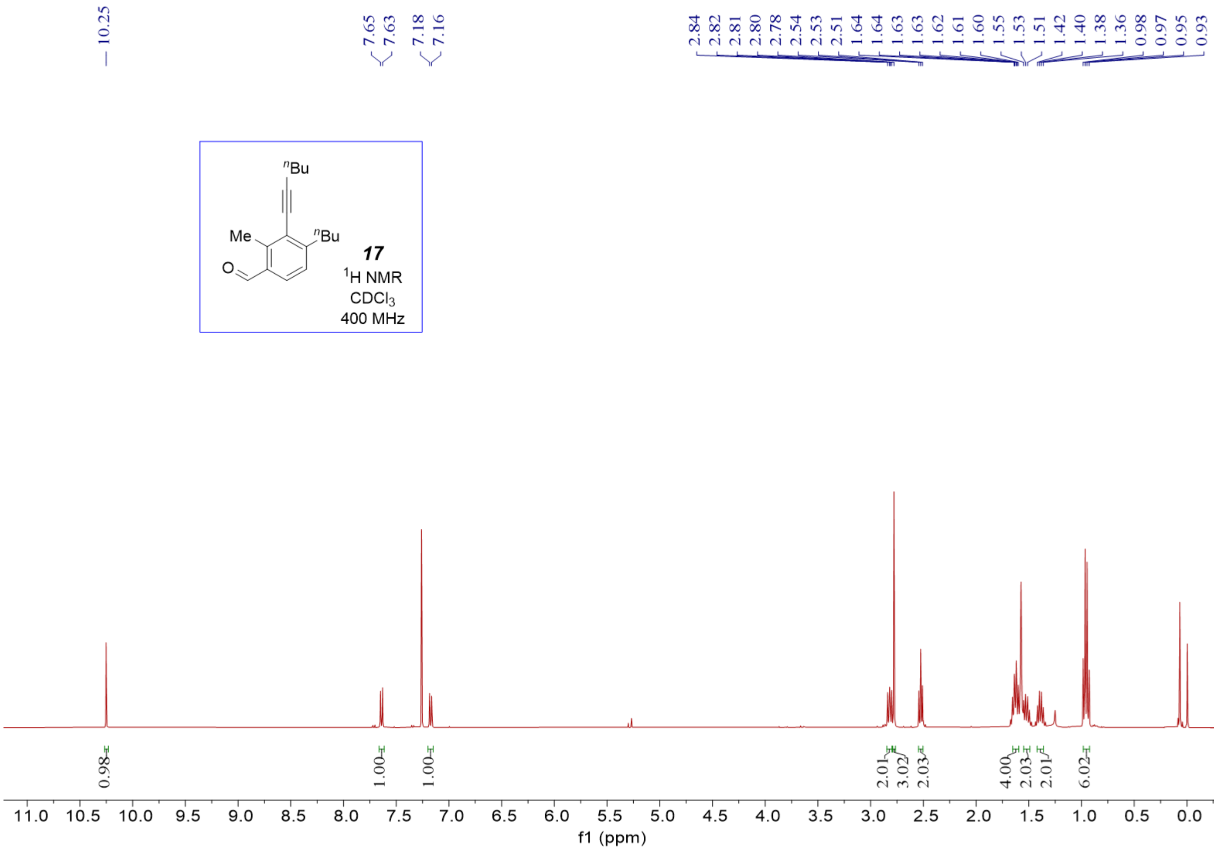

Figure S24 ^{1}H NMR Spectrum of Compound **17**

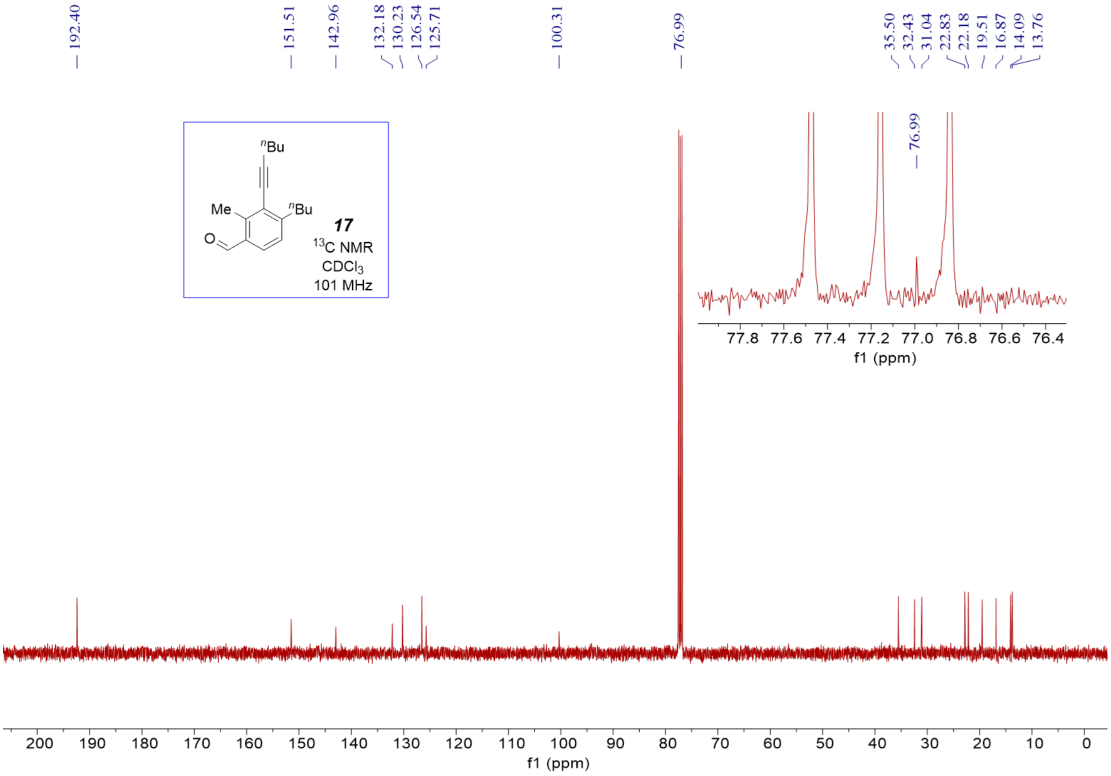

Figure S25 ^{13}C NMR Spectrum of Compound **17**

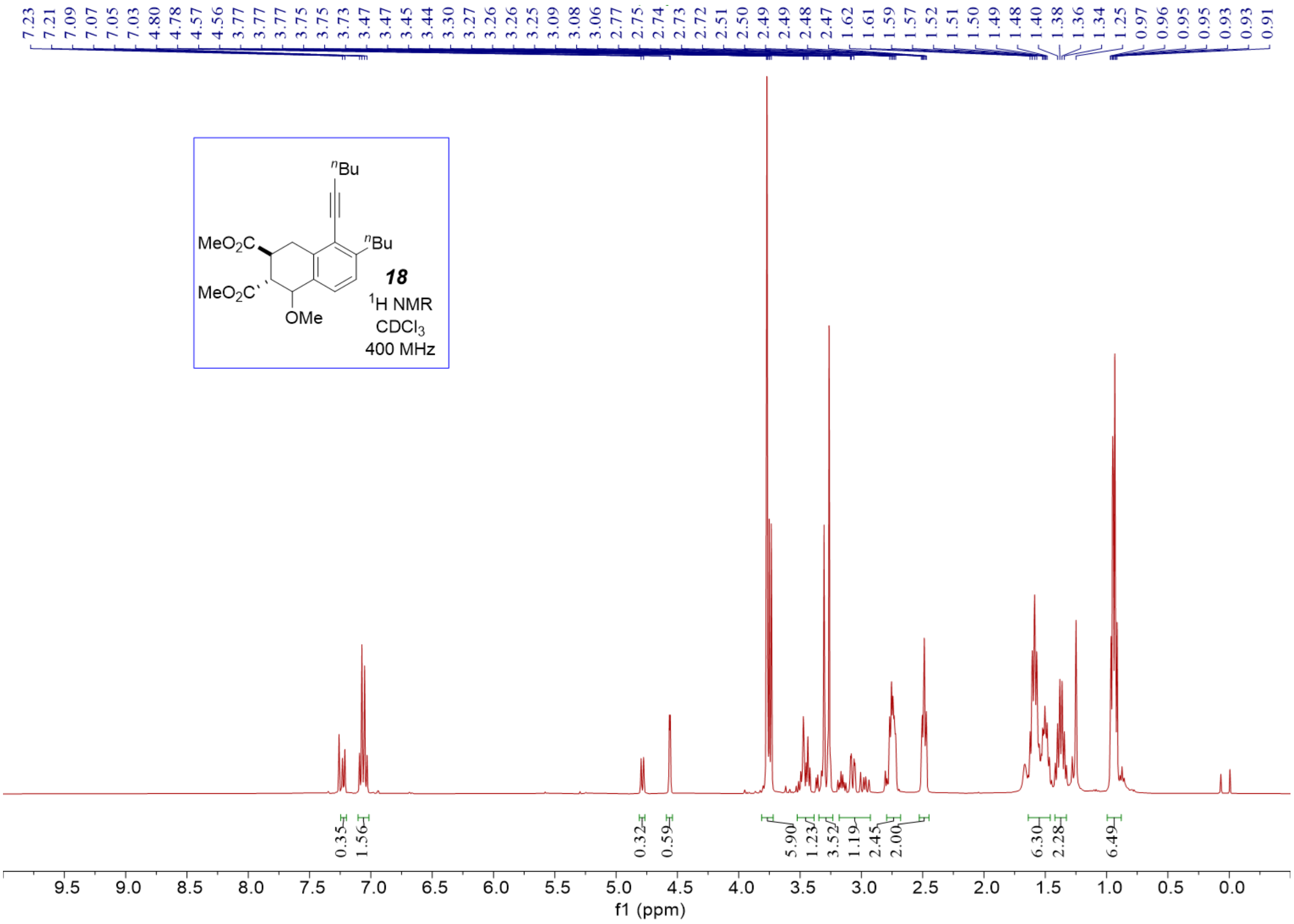

Figure S26 ^{1}H NMR Spectrum of Compound **18**

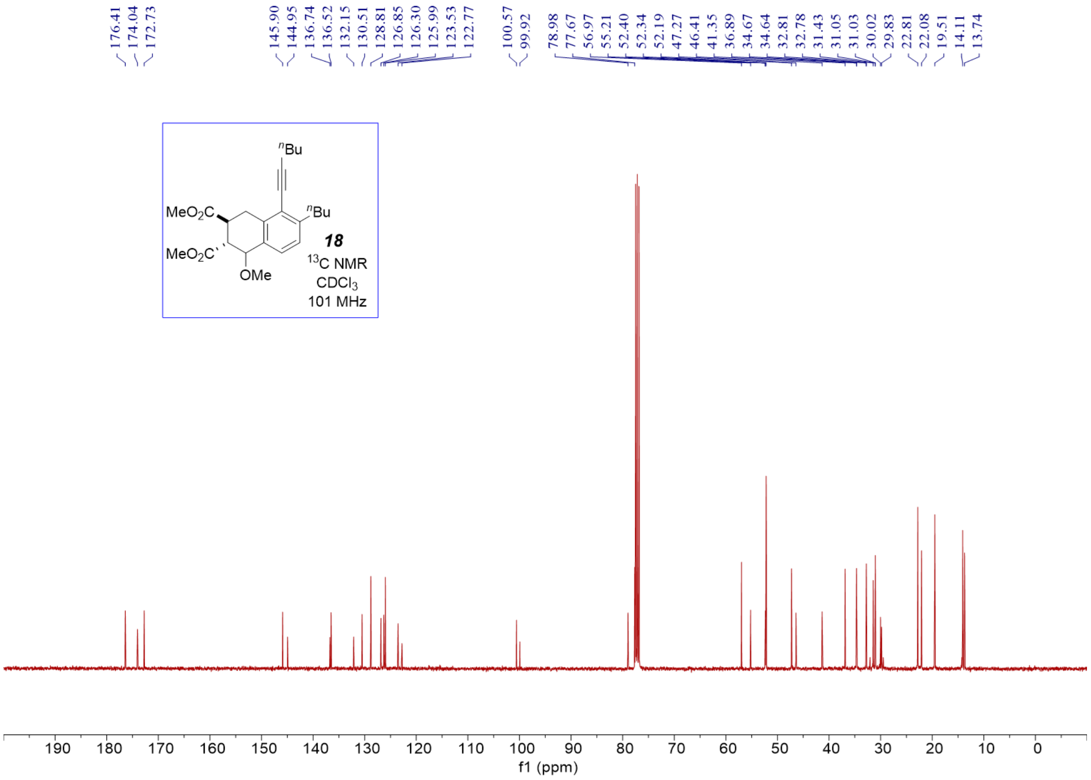

Figure S27 ^{13}C NMR Spectrum of Compound **18**
